# ﻿Taxonomic reappraisal of the European fauna of the bark beetle genus *Cryphalus* (Coleoptera, Curculionidae, Scolytinae)

**DOI:** 10.3897/zookeys.1179.101388

**Published:** 2023-09-08

**Authors:** Mathias Just Justesen, Aslak Kappel Hansen, Miloš Knížek, Åke Lindelow, Alexey Solodovnikov, Hans Peter Ravn

**Affiliations:** 1 Department of Geosciences and Natural Resource Management, University of Copenhagen, Rolighedsvej 23, 1958 Frederiksberg C, Copenhagen, Denmark; 2 Natural History Museum of Denmark, University of Copenhagen, Universitetsparken 15, 2100 København Ø, Copenhagen, Denmark; 3 Forestry and Game Management Research Institute, Strnady 136, CZ-252 02 Jíloviště, Czech Republic; 4 Department of Ecology, Swedish university of agricultural sciences, P.O. Box 7044, S–75007 Uppsala, Sweden

**Keywords:** *Cryphalusabietis*, *Cryphalusasperatus*, *Cryphalusdilutus*, *Cryphalusintermedius*, *Cryphalusnumidicus*, *Cryphaluspiceae*, C*ryphalus saltuarius*, dichotomous key, economic significance

## Abstract

Species in the genus *Cryphalus* are small and notoriously difficult to identify. Even among the relatively well studied European species, erroneous identifications are evident from literature and in museum collections. These misidentifications relate to the small size and similar appearance of *Cryphalus* species but they are also a product of insufficient diagnostic characters. This is especially unfortunate since some European species are considered pests. Based on the study of more than 1000 specimens and a thorough literature review, robust morphological and molecular evidence supporting all five hitherto recognised native species of *Cryphalus* in Europe is provided. A key for the reliable identification of these repetition species including new diagnostic characters recognised for the first time, including those from male genitalia, has been constructed. Each native species is provided with a detailed morphological description and their economic significance, distribution, and ecology discussed. Significant genetic variability is observed between certain clusters that should be further explored in a broader geographic context. Lastly, the need for a taxonomic revision of the genus *Cryphalus* for the entire Palearctic region due to the presence of many similar looking species which are often confused, thus distorting the knowledge of each species is highlighted.

## ﻿Introduction

*Cryphalus* Erichson, 1836 is the only genus of the scolytine tribe Cryphalini (Johnson et al. 2020a). According to Johnson et al. (2020a), it is defined by the combination of emarginated eyes, antennae with clearly visible sutures but without a septum, weakly bilobed third tarsal segments, proventriculus with a large apical plate, and the aedeagus with a sclerotised tegmen completing a ring and usually with two tegminal apodemes. Currently the genus includes 252 (Johnson et al. 2020b) + 1 species (Mandelshtam and Petrov 2022) distributed in Eurasia, Africa, Oceania, North America, Central America (introduced), and South America (introduced) (Johnson et al. 2020a). As far as known, all *Cryphalus* species feed on the phloem and cambium, and are monogamous. They create cave-like galleries under bark (Johnson et al. 2020a, b). A few species cause severe problems in loquat, fig, and mango production, and have therefore received taxonomic attention (Johnson et al. 2017, 2020b). However, the taxonomy and biology of the majority of *Cryphalus* species remains unknown.

In Europe, bark beetles are generally well studied. But even here, little systematic work has been done on the five species of *Cryphalus* hitherto known to be native in Europe. The *Cryphalus* species recorded from continental Europe include *Cryphalussaltuarius* Weise, 1891, *Cryphalusasperatus* (Gyllenhal, 1813), *Cryphaluspiceae* (Ratzeburg, 1837), *Cryphalusnumidicus* Eichhoff, 1878 and *Cryphalusintermedius* Ferrari, 1867. Additionally, the Asian species *Cryphalusdilutus* (Eichhoff, 1878) has been introduced to Malta (Mifsud and Knížek 2009), Italy (Faccoli et al. 2016), and France (Barnouin et al. 2020). Of these species only *C.dilutus* has received recent taxonomic attention (Johnson et al. 2020b), including high quality pictures of specimens, aedeagus, and proventricules.

Note that an application (Case 3832) has been sent to the International Commission on Zoological Nomenclature with the title “*Cryphalussaltuarius* Weise, 1891 (Coleoptera, Curculionidae): proposed conservation of the specific name by reversal of precedence with *Bostrichusasperatus* Gyllenhal, 1813 (currently *Cryphalusasperatus*)” (Justesen et al. in press A). If this reversal of precedence is accepted by the commission, *C.asperatus* effectively changes name to *Cryphalusabietis* (Ratzeburg, 1837). Additionally, *C.dilutus* was initially misidentified as *Hypocryphalusscabricollis* (Mifsud and Knížek 2009; Faccoli et al. 2016), a synonym of *Cryphalusdiscretus* Eichhoff, 1878 (Johnson et al. 2020a), which has led to some confusion about the presence of *C.discretus* in Europe.

The shortage of clear taxonomic diagnoses has led to many confusions and misinterpretations in literature on these relatively ‘well-known’ European species. This was highlighted in a paper by Eichhoff (1866), who questioned the validity of *C.abietis* (currently *C.asperatus*) described by Ratzeburg in 1837, as it seemed too similar to the species *C.saltuarius* (then known as *C.asperatus*), described by Gyllenhal in 1813. According to Eichhoff (1866), Ratzeburg himself even questioned the validity of his own species. The similarity between *C.piceae* and *C.numidicus* is also highlighted in the original description of *C.numidicus* (Eichhoff, 1878a) and exemplified by the key in Balachowsky (1949) that would lead any specimen of *C.numidicus* to *C.piceae*. Additionally, a study by Benhalima et al. (2005) used the name “*Cryphaluspiceaenumidicus*” in their study, again emphasising the similarity of these two species. Considering that the main diagnostic characters used today are the same as in the original descriptions from 1878, it is clear that new diagnostic characters are needed to separate these similar *Cryphalus* species found in Europe.

Even though genitalia have been successfully used to separate taxonomically difficult species within *Cryphalus* (Tsai and Li 1963; Zheng et al. 2019; Johnson et al. 2020a), they have not been studied in detail for the majority of *Cryphalus* species, including the European species. The only available illustrations of the European species are of *C.asperatus* and *C.dilutus* from a recently published paper by Johnson et al. (2020a), old genitalia drawings of *C.asperatus* by Ritchie (1918) and the same for *C.piceae* by Escherich (1923). Similarly, very few molecular barcodes are available from *Cryphalus* species, despite the fact that two recent studies (Zheng et al. 2019; Johnson et al. 2020b), successfully used barcoding as a tool to separate similar looking *Cryphalus* species. Most available *Cryphalus* sequences are from recent molecular phylogenetic work trying to solve the classification at higher taxonomic ranks, such as tribes or genera (Pistone et al. 2018; Johnson et al. 2020a) or from large scale barcoding projects with random inclusions of *Cryphalus* species. This fact is also reflected within the European species, where the number of publicly available sequences in the BOLD project (Ratnasingham and Hebert 2007) is currently restricted to 14 *C.asperatus* specimens, six *C.piceae*, six *C.saltuarius*, and zero for both *C.intermedius* and *C.numidicus*. Proventricules have been shown to differ between genera of bark beetles (Johnson et al. 2020a). Extracting proventricules is destructive and time-consuming, and therefore has some limitations, but as shown in Johnson et al. (2020b) it can be a useful character to separate *Cryphalus* species. To our knowledge only the proventriculus of *C.asperatus*, *C.dilutus* (Johnson et al. 2020a), and *C.piceae* (Escherich 1923) have been described. The proventricules of *C.saltuarius*, *C.intermedius*, and *C.numidicus* are undescribed.

As already mentioned, *Cryphalus* includes species capable of causing severe economic damage in the loquat, fig, and mango industry (Johnson et al. 2017, 2020b). *Cryphalusdilutus*, the species introduced to Europe, has also been reported to cause damage in figs (Faccoli et al. 2016). The five native European species are not regarded as serious pests, but *C.piceae*, *C.numidicus*, and to some extent *C.saltuarius* have been mentioned as bark beetles able to kill weakened trees. Both *C.piceae* and *C.numidicus* have been reported as problematic pests when the population density gets high (Toper 2002; Lieutier et al. 2016; Justesen et al. 2020). Despite this, most of the available biological data for the five species is scattered in smaller papers or older literature in several different languages often with a restricted focus.

The main motivation of this paper is to help guide a future Palearctic revision by highlighting the main disagreements in literature regarding the European species. An additional motive is to improve the diagnostic characters of the native European species, as the current characters can evidently lead to misidentifications, due to the very similar external morphology of *Cryphalus*. Lastly, we want to summarise the main bionomics of all five species, as this information could assist with species delimitation. In this contribution we aim to: 1) re-evaluate the current diagnostic characters of native European *Cryphalus*, including the critical and detailed examination of the male genitalia and proventricules of all five European species, and 2) implement DNA barcoding as a tool for delimiting these five European species, and lastly 3) review and summarise the available literature on the species known from Europe.

## ﻿Materials and methods

### ﻿Taxonomic procedures and terminology

We follow the morphological terminology used in the most recent review and reclassification of the tribe Cryphalini (Johnson et al. 2020a). The only difference in terminology is that we use setae instead of “bristles” and “hairs”, as we believe this to be a more accurate morphological term. To avoid lost characters and colour degradation in old museum specimens, as well as mislabelling, and problems with DNA extractions, we based our study on 1244 recently collected specimens from 15 European countries, shipped by various collectors (Table 1). This material was used for DNA extraction and morphological investigations. Additional material, not used for DNA extraction, is kept in 96% alcohol in a -20 °C freezer at the Natural History Museum of Denmark in Copenhagen. All mentioned synonyms were taken from the Palearctic cooperative catalogue (Alonso-Zarazaga et al. 2023).

**Table 1.** Examined material.

Species	No. of specimens	Country	Location	Coordinates	Collector
*Cryphaluspiceae*	22	Austria	Hummelbach	48.0763, 15.3627	M. Justesen
	8	Austria	Schönbuch	48.1661, 15.2657	M. Justesen
	5	Austria	Spitz	48.3585, 15.4040	M. Justesen
*Cryphalusasperatus*	23	Belgium	Momignies	49.9801, 4.1561	B. Moucheron
	7	Belgium	Bellefontaine	49.9100, 4.9700	B. Moucheron
*Cryphaluspiceae*	13	Belgium	Robechies	50.0900, 4.2700	B. Moucheron
*Cryphalusasperatus*	22	Czechia	Brdy	49.7500, 13.9600	M. Justesen
	1	Czechia	Silesia	49.9650, 18.1245	A./M. Knížek
	36	Denmark	Christiansfeld	55.3633, 9.4359	M. Justesen
	48	Denmark	Gisselfeld	55.2694, 11.9536	M. Justesen
*Cryphaluspiceae*	50	Denmark	Jyderup	55.6158, 11.4244	M. Justesen
*Cryphalusasperatus*	6	Denmark	Jyderup	55.6158, 11.4244	M. Justesen
	19	Denmark	Skærbæk	55.1700, 8.8400	M. Justesen
*Cryphaluspiceae*	14	Denmark	Skørping	56.8628, 10.0260	M. Justesen
*Cryphalusintermedius*	18	Germany	Dresden	51.0740, 14.4825	M. Justesen
*Cryphaluspiceae*	100	Germany	Baden-Württemberg	48.4000, 9.0000	H. Gebhardt
*Cryphalusnumidicus*	55	Greece	Leonidio	37.0560, 22.8124	M. Justesen
*Cryphaluspiceae*	113	Hungary	Sopron	47.6500, 16.4900	F. Lakatos
*Cryphalusasperatus*	98	Netherlands	Ameland	53.4541, 5.8068	T. Heijerman
*Cryphalussaltuarius*	72	Norway	Østby	63.0971, 11.6386	M. Justesen
	5	Norway	Sandvika	64.4600, 13.5700	Å. Lindelow
*Cryphalusasperatus*	6	Poland	Nowa Morawa	50.2331, 16.9253	M. Justesen
	6	Romania	Cacica	47.5891, 25.9275	N. Olenici
	23	Romania	Carlibaba	47.6016, 25.1933	N. Olenici
	6	Romania	Poiana Brașov	45.5969, 25.5669	N. Olenici
	31	Romania	Sucevița	47.7603, 25.6391	N. Olenici
	18	Romania	Cacica 2	47.6444, 25.8494	N. Olenici
*Cryphaluspiceae*	12	Romania	Cacica	47.5891, 25.9275	N. Olenici
	8	Romania	Cacica 2	47.6444 25.8494	N. Olenici
	3	Romania	Poiana Brașov	45.5969, 25.5669	N. Olenici
	1	Romania	Sucevița	47.7603, 25.6391	N. Olenici
*Cryphalusasperatus*	14	Slovakia	Bystrina	49.0318, 19.5911	Unknown
	44	Slovakia	Liptovský Mikuláš	48.9724, 19.5878	Unknown
*Cryphalussaltuarius*	3	Sweden	Björkvattnet	64.6000, 13.7700	Å. Lindelow
	6	Sweden	Gåddede	64.5000, 14.1300	Å. Lindelow
	12	Sweden	Strömsund	64.3600, 14.6400	Å. Lindelow
*Cryphalusasperatus*	5	Switzerland	Delemont	47.3729, 7.3291	M. Justesen
	187	Switzerland	Soyhières	47.3883, 7.3834	M. Justesen
*Cryphaluspiceae*	125	Switzerland	Delemont	47.3729, 7.3291	M. Justesen

### ﻿Photography and measurements

**Figure 1.**
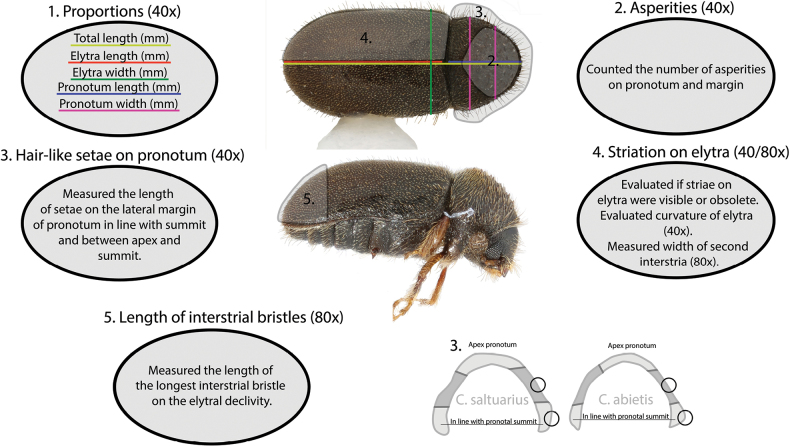
Morphological measurements and magnification at which they were measured.

Habitus images of all five species investigated here, and their diagnostic characters including genitalia, were taken using a Canon 5D Mark III camera with the Canon MP-E 65 mm 1–5× Macro Lens. Proventricule pictures were taken with a Canon 5D Mark III camera attached to a microscope (axioskop, Zeiss) with 400× magnification. Stacking was performed with the StackShot 3× Macro Rail with 20–25 photos stacked using the ‘PMax’ function in Zerene Stacker (v. T2020-05-22-1330). Post-processing of images was performed in Adobe Illustrator CC 2021 (v. 25.0.1) and Photoshop 2021 (v. 22.0.1). Editing was limited to the removal of background objects. All morphological observations and measurements were made using a LEICA M205C stereomicroscope (up to 160× magnification) with an ocular micrometre. In cases of large series, specimens for measurements were chosen based on a preliminary visual examination of the whole series to select individuals representing the entire size range. Specimens damaged or whose morphology was clearly affected by storage in alcohol were omitted from the measurements. For measurements, specimens from alcohol were first dried for minimum 30 min on paper towel and then placed in a glass Petri dish with fine sand to fix it in a desirable position needed for the measurements. To standardise the measurements, we made sure to focus simultaneously on the tubercles at the apex of pronotum and at the tip of the elytral declivity (ends of yellow line, Fig. 1). All measurements are given in millimetres (mm) or ratios. Characters to be measured were chosen based on characters used as diagnostic for species in nine identification keys (Reitter 1913; Ritchie 1918; Spessivtseff 1922; Balachowsky 1949; Stark 1952; Nunberg 1954; Hansen 1956; Grüne 1979; Pfeffer 1995; Noblecourt and Schott 2004). Additionally, new potential diagnostic characters were analyzed i.e., number of asperities, length of setae on lateral margin of pronotum, and proportions of proventricules and male genitalia. All our measurements were taken as shown in Fig. 1.

Note that the elytral width was measured below scutellum to ensure reproducibility (green line, Fig. 1), but some species might be slightly wider near the middle of elytra. In total we measured characters on 119 specimens distributed between the five species. These were then plotted using the ‘ggplot2’ package in R (v. 4.0.2) (R Core Team 2020).

### ﻿Dissections of genitalia and proventricules

Extraction of male genitalia was done by suspending the specimen in alcohol and then carefully removing the entire abdomen with a needle. The extracted abdomen was further cleared in a 10% KOH (Potassium hydroxide) aqueous solution for 30–90 minutes at room temperature depending on the degree of sclerotisation. Afterwards the tergites were removed, exposing the leftover muscle tissue surrounding the aedeagus. This muscle tissue was then carefully removed from the sclerotised aedeagus with a thin needle. Proventricules were extracted using the same technique. The cleaned aedeagi and proventricules were photographed using the same imaging setup described in the section above. Based on these images and visual examinations, we used Adobe Illustrator to create schematic drawings of the genitalia which would stress diagnostic characters in the best way. Due to the minute size of the specimens and their fragile sclerites, the extraction of genitalia often resulted in damaged or moved parts, e.g., the tegmen and/or aedeagus apodemes. To overcome this and ensure that potential intraspecific variation was accounted for, aedeagi were extracted from six specimens of each species. However, because of the small number of *C.intermedius* specimens available, and most of them being females, we only studied one *C.intermedius* aedeagus. Based on the morphological measurements obtained, we constructed an identification key based on external characters and where useful added the species-specific characters of the male genitalia.

### ﻿Scanning electron microscopy

For SEM examination specimens were mounted on aluminium stubs with flexible aluminium tape, then coated with platinum/palladium and studied in a JEOL JSM-6335F scanning electron microscope.

### ﻿Molecular analysis

To confirm the validity of the five *Cryphalus* species investigated in this study, we sequenced mitochondrial cytochrome c oxidase subunit I (COI) from all five species. DNA extraction was done using an in-house protocol. Firstly, the entire specimen was crushed in an Eppendorf tube with beads on a Retsch MM400, with settings 25 pr. 1/s. Then 80 µl lysis buffer was added to the sample and the step above repeated. The sample was then centrifuged (14000 RPM) on an Eppendorf 5810 centrifuge for 2 min and left at 65 °C for 2 hours. 160 µl, 2× MagNa (Magnetic bead mix) was added to the sample and then placed on a magnet rack for 3 min, afterwards the supernatant was removed. 150 µl 80% alcohol was added to the sample (while still on the magnet rack) and gently circulated with the pipette. The supernatant was removed. This step was repeated; however, the second time the sample was left to dry for a few minutes to ensure all traces of alcohol were removed. The sample was removed from the rack and 30 µl 0.1× TE-buffer was added. Then the sample was left for 10 min at 56 °C. Again, the sample was placed on the magnet rack, and the supernatant was transferred to an Eppendorf tube for PCR (Polymerase Chain Reaction). PCR of COI was done on the extracted DNA with the following protocol: 12.5 µl mastermix (Phire Plant direct PCR Master Mix) was mixed with 0.5 µl of each primer (LCO1490 and HC02198; Folmer et al. 1994), 10.5 µl denatured H_2_O and 1 µl extracted DNA.

Reactions were amplified on a BIO RAD T100 thermal cycler. Samples were heated and kept at 98 °C for five min following 35 cycles of: 7 s at 98 °C, 7 s at 54.3 °C, and 20 s at 72 °C, followed by a final extension step at 72 °C for one minute. Amplifications were confirmed by standard gel electrophoresis. PCR products were sent to Eurofins (Konstanz, Germany) for sequencing.

All generated and previously published sequences of *Cryphalus* species were imported to Geneious Prime (v. 2022.2.2). Sequences of *Cryphalus* species in conifers and the species most closely related to the five target species were kept in the final species tree, the rest were omitted. Sequences were then aligned using the MAFFT Multiple Alignment plugin (v. 1.5.0) based on MAFFT (Katoh et al. 2002). To calculate average intraspecific distance and interspecific distance to nearest neighbour we used the Species Delimiter plugin (Masters et al. 2011) as implemented in Geneious. The full 658 bp alignment was partitioned by codon position and imported to ModelFinder (Kalyaanamoorthy et al. 2017). We then ran two separate phylogenetic analyses one using partition and substitution model recovered in ModelFinder. First a Maximum Likelihood (ML) analysis using IQ-Tree (v. 1.6.10) (Nguyen et al. 2015) with default settings except: ultrafast Bootstrap (UFP) was run for 1000 iterations (-bb 1000), then re-run with up to 10,000 iterations (-nm 10,000) with SH-aLRT test (-sh_test true). Second a Bayesian analysis (BI) using MrBayes (v. 3.2.7a) (Ronquist et al. 2012) consisting of two runs of four chains each, with default settings except that different rates of evolution were allowed for each partition (ratepr = variable). Convergence of each analysis was examined by checking the Potential Scale Reduction Factor (PSRF) in Tracer v. 1.7.1 (Rambaut et al. 2018). For each analysis we considered posterior probability values (PP) ≥ 0.90, SH-aLRT ≥ 80, and UFB ≥ 95 to indicate clade support.

### ﻿Assembly of data on bionomics

To further characterise species and highlight biological differences between them, we gathered any available bionomic information about each of them. Most information was found in literature and supplemented based on our own field experiences. Based on data from Wermelinger et al. (2002), we also evaluated the flight activity of adult beetles.

### ﻿Assembly of data on distribution

We summed up species distributions according to the recent palearctic catalogues (Knížek 2011; Alonso-Zarazaga et al. 2023) and discussed it in the respective sections for each species. An estimate of the distribution was created for each species. Due to possible mix-ups with similar looking species in certain regions, geographical areas of interest were highlighted on the maps. The created maps were made in the online application SimpleMappr (Shorthouse 2010) with additional work in Adobe Illustrator.

## ﻿Results and discussion

### ﻿Morphology

**Figure 2.**
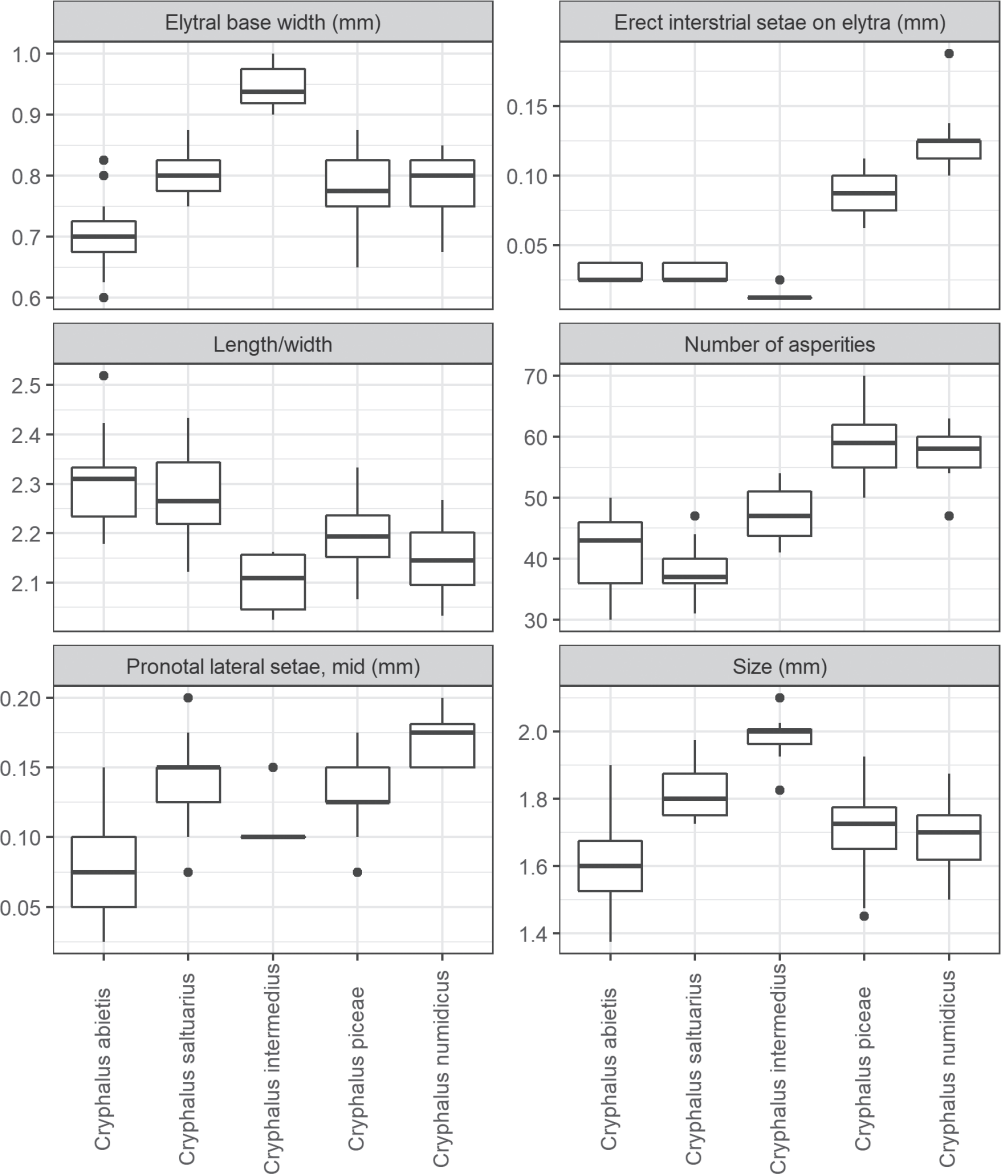
The variation of measured characters in five European *Cryphalus* species. Pronotal lateral setae, mid (mm), refers to the length of the lateral setae on pronotum between the summit and the apex.

**Figure 3.**
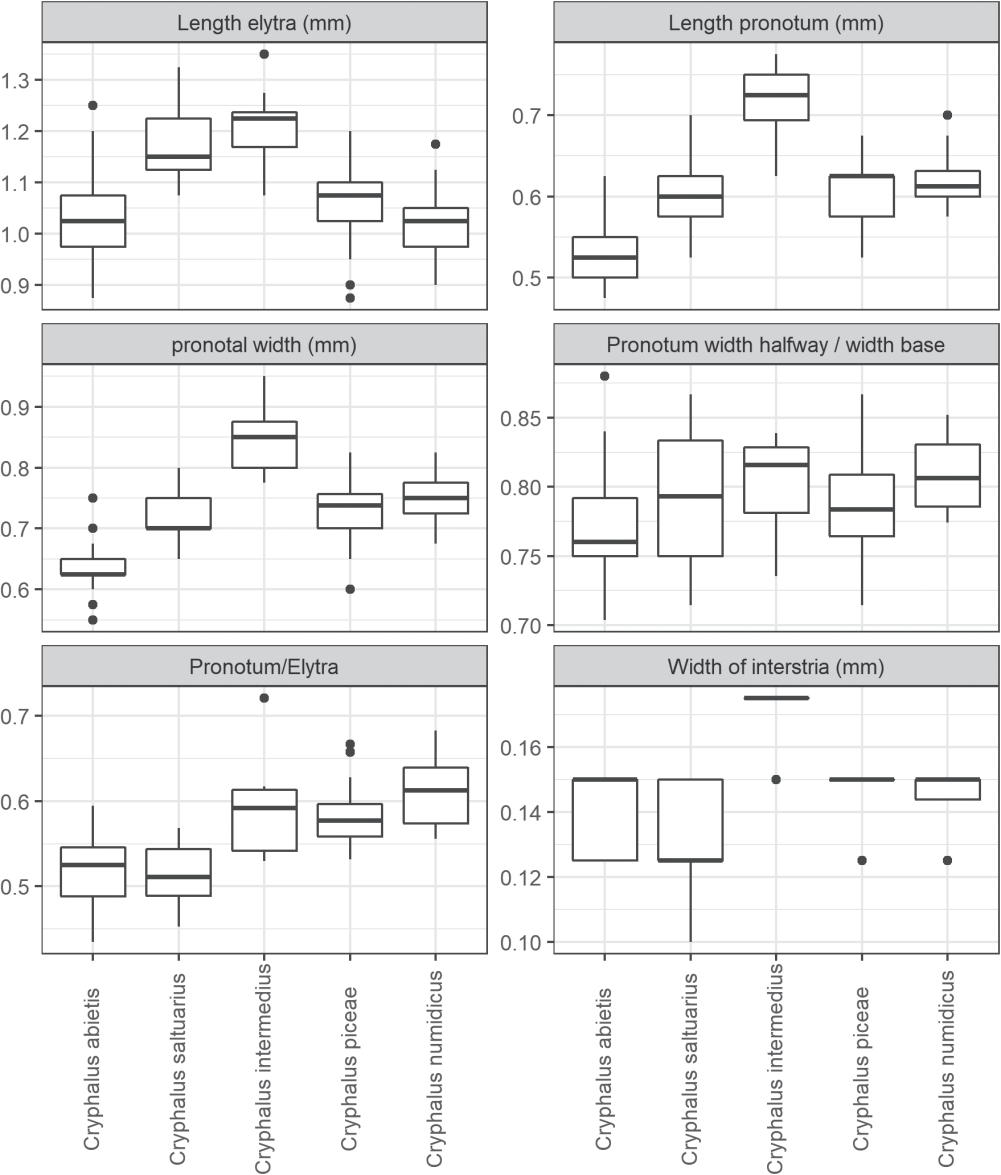
The variation of measured characters in five European *Cryphalus* species.

**Figure 4.**
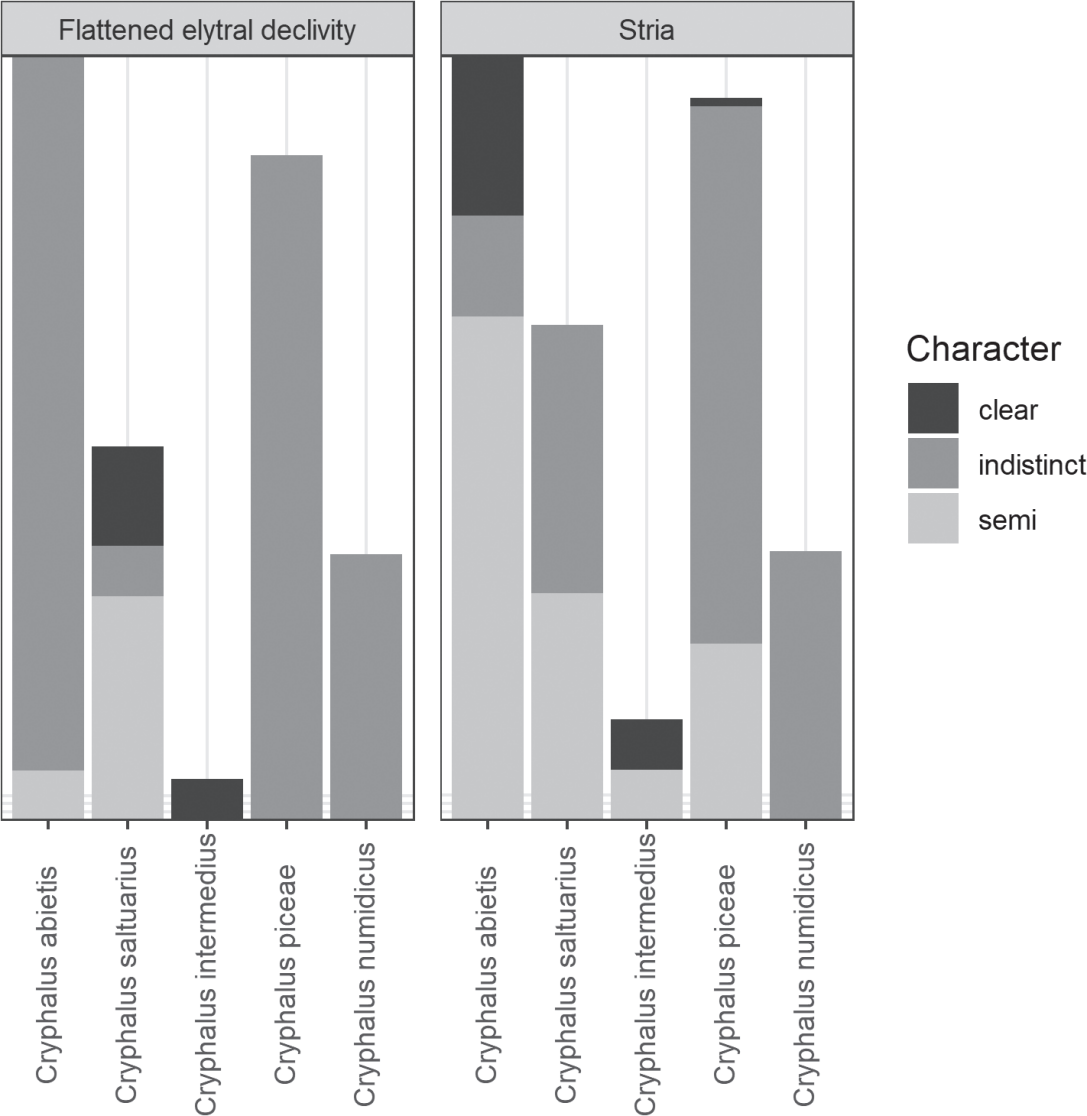
The variation of measured characters in five European *Cryphalus* species.

The results of measurements can be seen in Figs 2–4. Generally, they show overlap when comparing all five species, but between single species, several characters had little or no overlap. No specimens of *Cryphalusdilutus* were analysed, but the species is described in Johnson et al. (2020b). Characters separating the species-pairs *C.piceae*/*C.numidicus* and *C.asperatus*/*C.saltuarius* from each other were established, but separating *C.piceae* from *C.numidicus*, and *C.asperatus* from *C.saltuarius*, was difficult. All investigated morphological characters used to separate these two species-pairs overlapped between the species, although by little in some cases.

There was overlap in the sizes of the five species, but usually *C.intermedius* was longer followed by *C.saltuarius* and *C.piceae*/*C.numidicus* (Fig. 2). In contrast, *C.asperatus* was the shortest species, with little overlap in size with *C.intermedius*/*C.saltuarius*. Part of the size overlap between *C.saltuarius* and *C.asperatus*, was explained by a single *C.asperatus* individual which was significantly larger than the others. This individual was verified as *C.asperatus* using the molecular methods described above.

When looking at width parameters, *C.intermedius* was the widest species. Especially the width of elytra clearly separated *C.intermedius* from the remaining species (Fig. 2), but also pronotum and interstriae were comparably wider in *C.intermedius* (Fig. 3).

When comparing length/width proportions (Fig. 2) it was clear that *C.asperatus*/*C.saltuarius* was slimmer and longer compared to the more stout body shape of especially *C.intermedius* but also *C.piceae* and *C.numidicus*. Additionally, *C.numidicus* was slightly stouter than *C.piceae*, but this character had large overlap between the two species. When looking at the proportion of pronotum length to elytra length (Fig. 3), *C.asperatus* and *C.saltuarius* had a short pronotum relative to elytra compared to *C.piceae*/*C.numidicus*/*C.intermedius*, but these proportions overlapped when including the variability within species.

Two characters with little or no overlap separating the species pairs *C.piceae*/*C.numidicus* and *C.asperatus*/*C.saltuarius* were established. The length of declivital interstrial setae was longer in *C.piceae*/*C.numidicus* and did not overlap with *C.asperatus*/*C.saltuarius*/*C.intermedius*. Additionally, the length of interstrial setae was usually longer in *C.numidicus* compared to *C.piceae*, but with overlap. It is the authors′ experience from previous studies collecting living *C.piceae*, that a few outlier specimens can have markedly shorter setae (perhaps abraded), overlapping in length with *C.asperatus*/*C.saltuarius*. The number of asperities had very little overlap between the species pairs *C.asperatus*/*C.saltuarius* and *C.piceae*/*C.numidicus*, but the latter pair of species almost always had > 50 asperities (Fig. 2). *Cryphalusintermedius* was found to be intermediate between the two species pairs.

The length of the lateral setae on pronotum between summit and apex (Fig. 2) overlapped between the five species, but especially between *C.asperatus* and *C.saltuarius*, there was only little overlap, with generally shorter setae between summit and apex in *C.asperatus* compared to *C.saltuarius* and the other three species.

The declivity tended to be more flattened on *C.saltuarius* and *C.intermedius* (Fig. 4). There was some overlap between *C.saltuarius* and *C.asperatus*, as some *C.asperatus* specimens had a slightly flattened declivity, overlapping with *C.saltuarius* specimens with a less clearly flattened declivity. The distinctness of striae was a difficult character to score, also reflected in the data (Fig. 4). There was a tendency of *C.asperatus* and *C.intermedius* having more distinct striae, but there was a large variation between and within species.

Part of the overlap between the species could be explained by a varying degree of reaction to storage in alcohol, protruding the head in different angles or variation in swelling of the specimens. Additionally, differences in the placement of wings, limbs, or head at the time of death created variation between specimens. Although we obtained measurement in the most standardised way possible, some variation has unavoidably been introduced, especially due to the small size of the investigated species.

**Figure 5.**
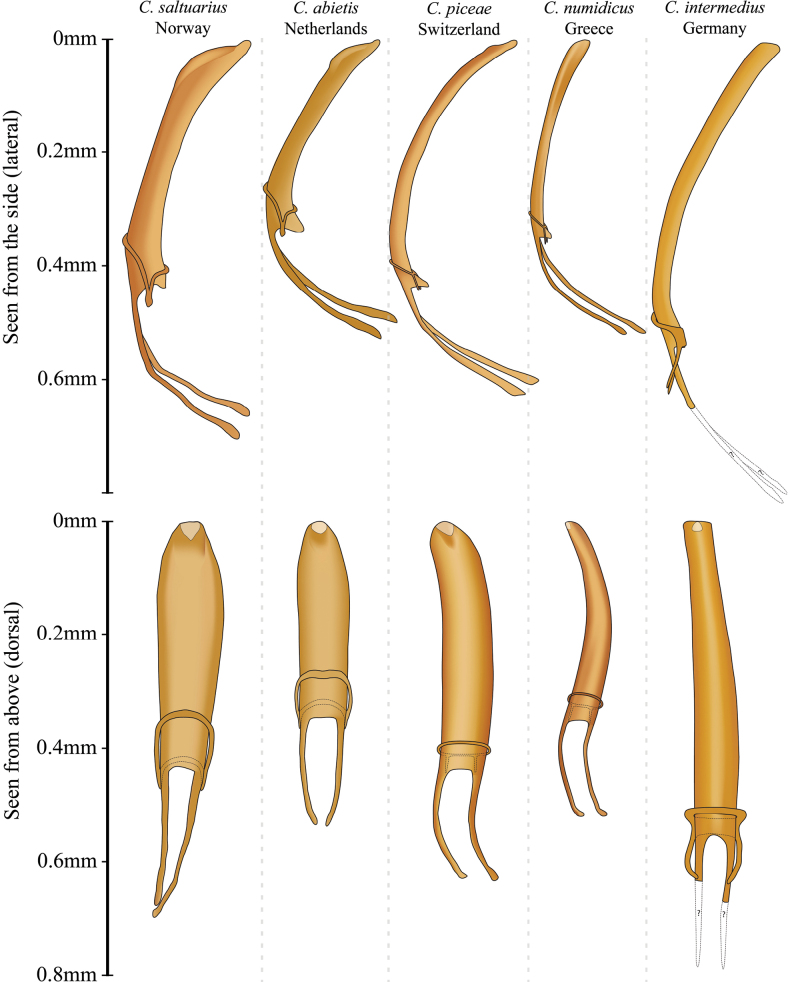
Lateral and dorsal illustration of male genitalia of five European *Cryphalus* species.

For all five species, the extracted aedeagi are clearly distinguishable (Fig. 5) (for images of aedeagi, see Suppl. material 1). Morphologically, the aedeagi of *C.asperatus* and *C.saltuarius* are similar, and the same for *C.piceae* and *C.numidicus*. The aedeagus of *C.intermedius* did not resemble any of the other aedeagi.

**Figure 6.**
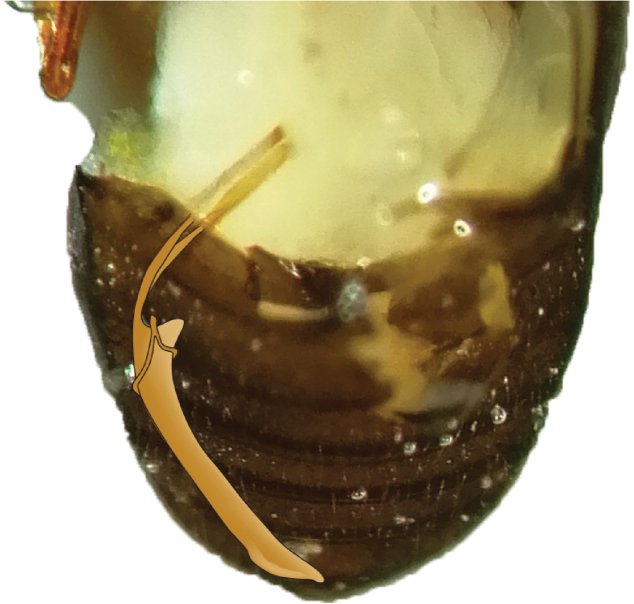
Placement of aedeagus in *Cryphalus* spp. when viewed dorsally with the wings removed.

We found that the aedeagus was positioned on the right side of the abdomen when viewed ventrally (Fig. 6), a slightly different placement than is depicted in Johnson et al. (2020a: fig. 5). This could not be confirmed for *C.intermedius*, as we had too few specimens, but we expect it to be positioned similarly to the other species.

**Figure 7.**
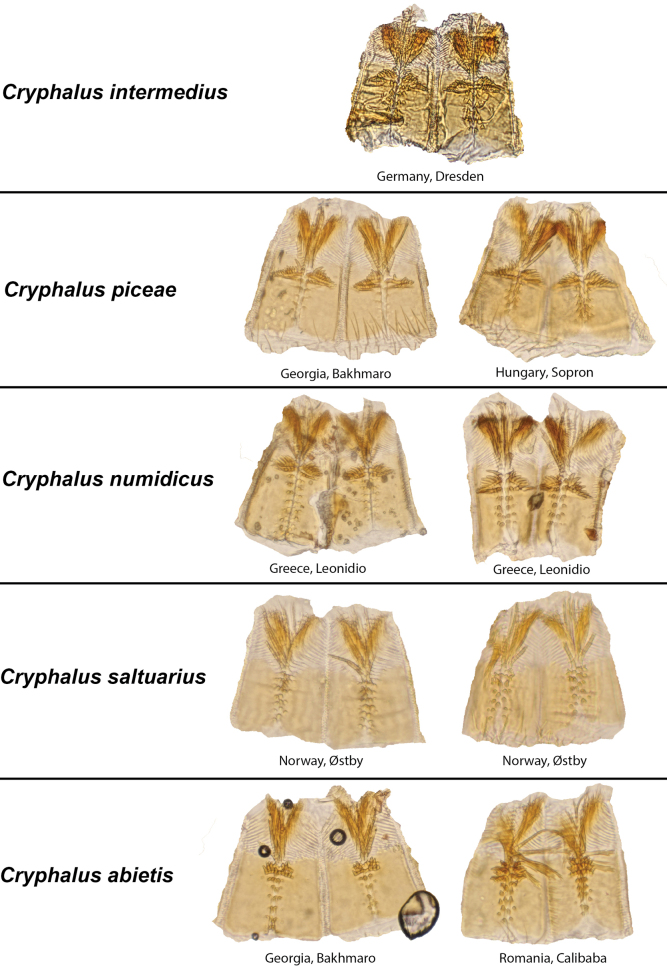
Proventricules of the five native *Cryphalus* species currently recognised in Europe.

Proventricules were useful in separating *C.asperatus* and *C.saltuarius*, but we could not separate *C.piceae*, *C.numidicus*, and *C.intermedius*, based on the shape of the proventricules (Fig. 7).

### ﻿Molecular analysis

**Figure 8.**
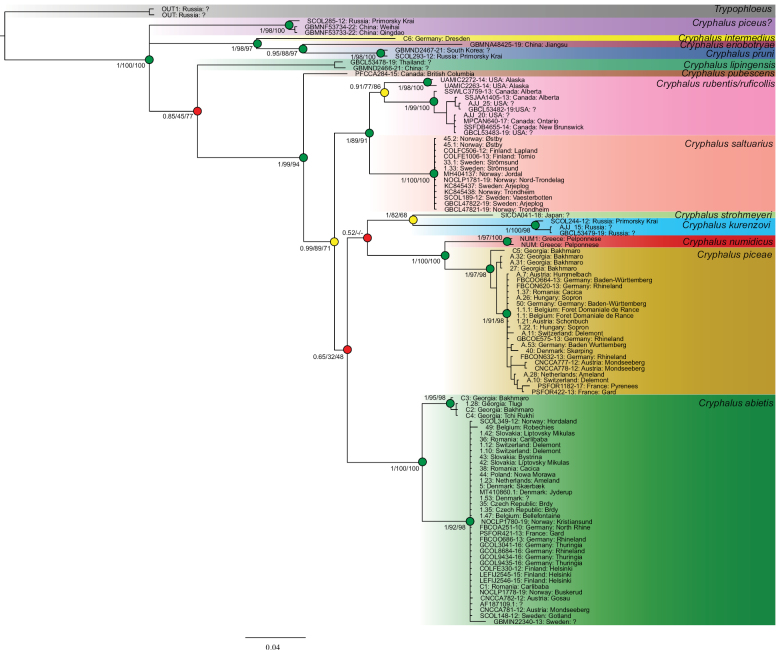
Species tree based on COI barcodes. Support values are given as posterior probability for Bayesian analysis and ultrafast bootstrap and SH-aLRT values for maximum likelihood analysis. Nodes coloured green for PP ≥ 0.90, UFB ≥ 80 and SH-aLRT ≥ 95, yellow for either PP ≥ 0.90, UFB ≥ 80 or SH-aLRT ≥ 95, and red for PP < 0.90, UFB < 80 and SH-aLRT < 95.

We obtained COI sequences from all five European species (Fig. 8).

Three separate lineages were found. The first includes three specimens from China and Far East Russia. Two (GBMNF53733-22 and GBMNF53734-22) were recently published (Chang et al. 2021) and associated in BOLD with the last member SCOL285-12 identified as *C.piceae*. They used this wrongly identified specimen as verification, so even though neither match the DNA barcode of true *C.piceae* this identity was used. This has caused an association with an ophiostomatoid fungus to be wrongly recorded, highlighting the need for better verification of databases. We have been unable to obtain any of these specimens for validation, but believe that the correct species may be *Cryphaluspiceus* Eggers, 1926 which is known from that region. The second lineage includes the European representative *C.intermedius* together with *C.eriobotryae* Johnson, 2019, and *C.pruni* Eggers, 1929 from China, South Korea, and Far East Russia. Lastly the remaining members were found in a single lineage.

Comparisons of COI sequences (Fig. 8), show that the American species, *C.ruficollis* Hopkins, 1915 and *C.rubentis* Hopkins, 1915 are more similar to *C.saltuarius* (9.6% divergence), than *C.saltuarius* is to *C.asperatus*. This was supported by observed morphological similarities between *C.saltuarius* and *C.ruficollis*/*rubentis*.

*Cryphaluspiceae* and *C.numidicus* were more similar to each other (9.0%) than to *C.strohmeyeri* Stebbing, 1914 and *C.kurenzovi* Stark, 1936 specimens. The *C.kurenzovi* specimen is also clearly different from both *C.strohmeyeri* (14.4%) and the clade with *C.numidicus* and *C.piceae* (21.8%). This is an interesting find, considering that according to Wood (1992a) Eggers synonymised *C.kurenzovi* and *C.piceae*, as they too are, at least superficially, morphologically similar. Today *C.kurenzovi* is considered a valid species (Johnson et al. 2020a). Furthermore, we found a slight genetic difference (1.5%) between *C.piceae* from Georgia and those from the remaining sampled areas.

For *C.asperatus*, four specimens from two localities in Georgia were found as sisters to the remaining *C.asperatus* members, with a distance of 5.6% and high support. Broader geographical sampling in and around Georgia, including in-depth morphological study, could help elucidate the relationship of these and establish if they represent a separate species or intraspecific variation of *C.asperatus*. Initial studies reveal no morphological differences in adeagi, proventricules or the other measured characters.

### ﻿Neotypes

No name-bearing type specimens exist for *C.piceae*, *C.asperatus*, *C.intermedius* and *C.numidicus*. The authors consider that it is necessary to designate name-bearing types for *C.piceae*, *C.asperatus*, and *C.numidicus* to define the nominal taxon objectively. This is due to the many taxonomically similar species within the currently recognised range of these three species. So, to account for future *Cryphalus* studies, we designated neotypes of *C.piceae*, *C.asperatus*, and *C.numidicus*. Preferably neotypes should have an associated DNA sequence, as this will prove useful for future taxonomic work on *Cryphalus*. This is the case for conspecifics of *C.numidicus* and *C.piceae*, and will be added to the *C.asperatus* neotype in the near future. Additionally, all neotypes are males, pinned with an extracted aedeagi, to ease comparisons in future studies. All specimens are deposited at the Natural History Museum of Denmark (NHMD). Details of the neotype designations can be seen in the relevant species sections below.

### ﻿Bionomics and distribution

All five native European species are phloem feeders and have a preference towards recently broken branches or otherwise fresh but weakened material (pers. obs. MJJ). The number of generations a year, overwintering strategy, and phenology varies depending on the species and locality. Data from Wermelinger et al. (2002) combined with available literature is used for discussion in each species section. None of the five species can be regarded as serious pests, but *C.piceae*, *C.numidicus*, and to some extent *C.saltuarius* have been reported attacking weakened trees. Distributional overlap between species is poorly investigated in especially the East Palearctic, but also in the countries around the Levantine Sea, and in the Caucasus. In a future Palearctic revision, we suggest adding distributional data from specimens at museums and in private collections, to elucidate the actual distribution. Krivolutskaya (1996) and Johnson et al. (2020b) provide a good overview on the diversity of *Cryphalus* present in the east Palearctic. Biology, harmfulness, distribution, and taxonomy is further discussed in each species section below.

### ﻿Key to the European *Cryphalus* species

**Table:** 

1	Pronotal disc covered by scale-like setae; frons in females simple, convex, in males with straight transverse carina above the level of eyes; mesofemur simple in females, with spur in males	***C.dilutus* Eichhoff, 1878**
–	Pronotal disc covered by hair-like setae; frons simple, convex in both sexes; mesofemur simple in both sexes	**2**
2	Erect elytral interstrial setae at least as long as the width of second interstria, well visible. Asperities (47–70) in concentric circles on pronotal declivity	**3**
–	Erect elytral interstrial setae shorter than width of second interstria. Randomly distributed asperities (30–54) on pronotal declivity	**4**
3	Erect elytral interstrial setae 0.13–0.23 mm long, same length or only slightly longer than width of second interstria. Pronotum anteriorly slightly constricted. Usually less hairy appearance than *C.numidicus*. Penis body in dorsal view only slightly spirally twisted, > 0.4 mm	***C.piceae* (Ratzeburg)**
–	Erect elytral interstrial setae 0.20–0.38 mm long, clearly longer than width of second interstria. Pronotum almost circular. Generally more hairy appearance than *C.piceae*. Penis body in dorsal view distinctly spirally twisted, < 0.4 mm	***C.numidicus* Eichhoff**
4	Body length usually > 1.93 mm (1.83–2.10 mm). Elytral width > 0.9 mm (0.9–1 mm), elytral striae visible on elytral declivity. Penis body > 0.5 mm, tegminal apodemes ~ 2× the length of the distance between them	***C.intermedius* Ferrari**
–	Body length usually < 1.93 mm (1.38–1.98 mm). Elytral width < 0.9 mm (0.6–0.88), elytral striae indistinct on elytral declivity. Penis body < 0.5 mm, tegminal apodemes ~ 1/2 the length of the distance between them	**5**
5	Body length usually < 1.75 mm (average 1.61 mm). Elytral striae often clear, with discal striae deeper than those on elytral declivity (degree of striation varies among specimens). Elytral declivity often with regular curvature. Lateral setae on pronotum in line with summit clearly shorter than setae between summit and apex. Penis body in dorsal view, except at apex, equally broad along its length. Entire aedeagus ~ 0.5 mm long	***C.asperatus* (Gyllenhaal)**
–	Body size usually > 1.75 mm (average 1.82 mm). Elytral striae often obscure (individual specimens with more or less clear striae). Elytral declivity often slightly flattened in the middle. Lateral setae on pronotum in line with summit same length, or only slightly shorter than setae between summit and apex. Penis body in dorsal view broadest one quarter down from the apex and then becomes increasingly narrowed towards the base. Entire aedeagus > 0.6 mm	***C.saltuarius* Weise**

### ﻿Descriptions

#### ﻿ Cryphalus piceae

Taxon classificationAnimaliaColeopteraCurculionidae

(Ratzeburg, 1837)

8AFDF89B-5D38-5945-87FF-C3BA4C9EF155

Cryphalus orientalis Eggers, 1911b: 122 (syn: Pfeffer and Knížek 1993).
Cryphalus hattorii Kôno, 1938: 67 (syn: Inouye and Nobuchi 1957).
Cryphalus subdepressus Eggers, 1940d: 37 (syn: Wood 1992a).

##### Type material.

According to Wood (1967) and Horn et al. (1990b), Ratzeburg’s material was destroyed during WWII. The authors have confirmed that the material was not present at the listed museums in Horn et al. (1990B), and it is therefore presumably destroyed.

##### Neotype designation.

**Figure 9.**
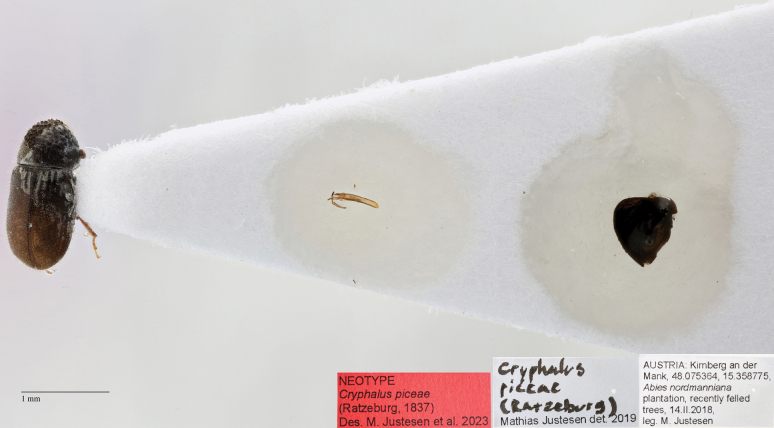
Neotype of *Cryphaluspiceae*, stored at NHMD in the entomological collections.

We designate a neotype of *Cryphaluspiceae* with the express purpose of clarifying the taxonomic status. The original description was based on specimens collected either in Upper Silesia (Poland) or Bavaria (Germany) (Ratzeburg 1837). A neotype of *Cryphaluspiceae* (Ratzeburg, 1837) was designated (Fig. 9). It is a male collected 14/02-2018 in Austria (48°04'31.3"N, 15°21'31.6"E) from an *Abiesnordmanniana* (Steven) Spach branch, not far from Bavaria. The specimen will be stored at NHMD in the entomological collections. COI sequence (Fig. 8; A.7 Hummelbach) is from a specimen collected in the same branch as the neotype.

##### Material examined.

474 specimens from various locations in Europe (Table 1) were examined. Morphological measurements were made on 33 specimens from Austria (7), Germany (12) and Hungary (14). The average results are shown in Fig. 2.

##### Diagnosis.

**Figure 10.**
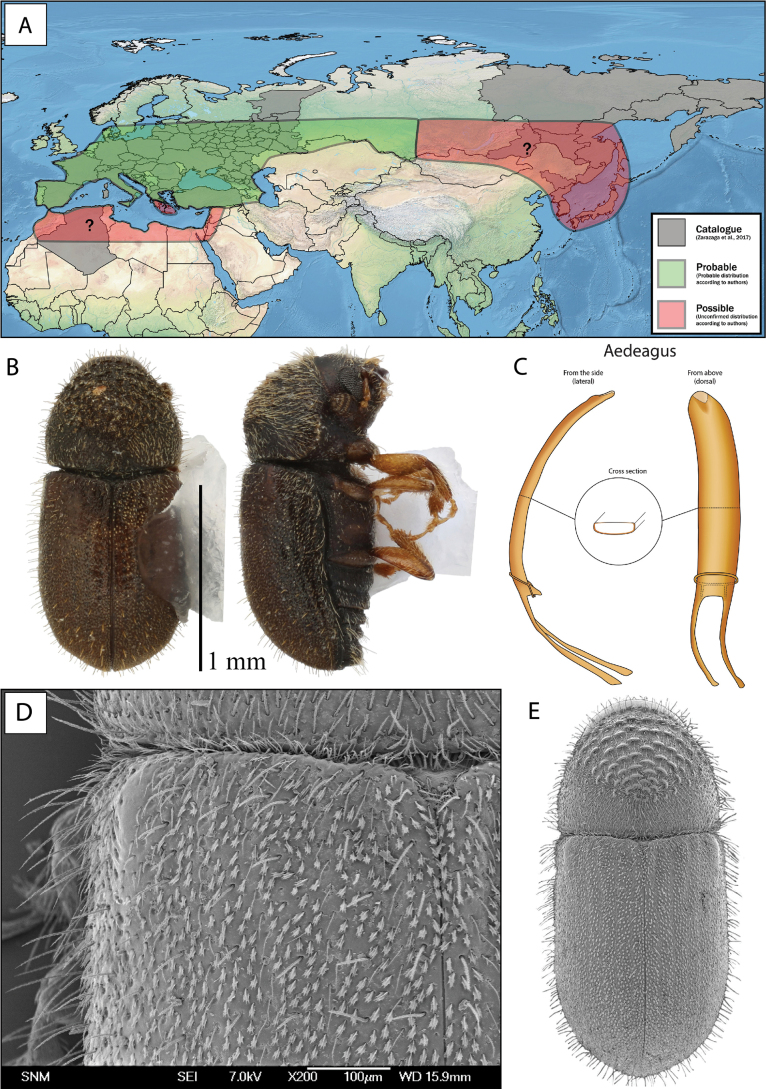
*Cryphaluspiceae***A** distribution **B** lateral and dorsal view **C** aedeagus **D** SEM, specimen from Sopron, Hungary **E** SEM (43× magnification), specimen from Sopron, Hungary.

This species can be diagnosed from morphologically similar *Cryphalus* in Europe by the combination of a circular pronotum that is anteriorly constricted, asperities (> 50) on pronotum in almost concentric circles, long erect interstrial setae on the elytral declivity approximately same length or only slightly longer than width of second interstria. For confident identification the male genitalia is unique. The penis body when seen from above (dorsally) is equally broad and asymmetric, slightly spiralled. The entire aedeagus is ~ 0.6 mm in length (Fig. 10B–E).

##### Description.

Length 1.45–1.93 mm, average size 1.73 mm (neotype 1.85 mm). ***Proportions*** 2.21× as long as wide, elytra 1.35× as long as wide, elytra 1.72× as long as pronotum. ***Antennae***: club with three procurved sutures marked by coarse and long setae. Funiculus with four antennomeres (with pedicel). ***Pronotum***: dark brown to black on both slope and disc. Profile anterior to summit rounded but slightly constricted anteriorly, wider in line with summit. Anterior margin with 4–8 asperities, the outer one or two pairs usually smaller; erect setae on entire lateral margin of pronotum. Anterior slope with > 50 asperities, including the ones on the anterior margin. Disc ~ 1/4 the length of pronotum, gently sloped, weakly tuberculate surface texture with small hair-like setae in each tubercule. Vestiture on pronotal declivity and disc hair-like. Suture between pronotum and elytra weakly sinuate. ***Scutellum***: with trifurcate setae on the margin towards elytra (only visible at high magnification). ***Elytra***: usually brown to black, if brown often darker at base, sometimes well-developed adults are light brown, elytral margins slightly wider 2/3 from base. Elytral declivity regularly rounded. Surface smooth. Striae with rows of punctures, each puncture with a short hair-like seta, punctures sometimes visible. Interstrial setae long (0.13–0.23 mm) and erect. Interstrial ground vestiture (scales) are serrated, ~ 2–3× as long as wide and translucent brown with a weak iridescence (Fig. 10B, D, E). ***Proventriculus***: sutural teeth of irregular size, confused, in two or more longitudinal rows. Apical teeth extend laterally over the entire segment. Masticatory brush slightly < 1/2 of the proventricular length (Fig. 7). Proventriculus also illustrated in Escherich (1923).

**Figure 11.**
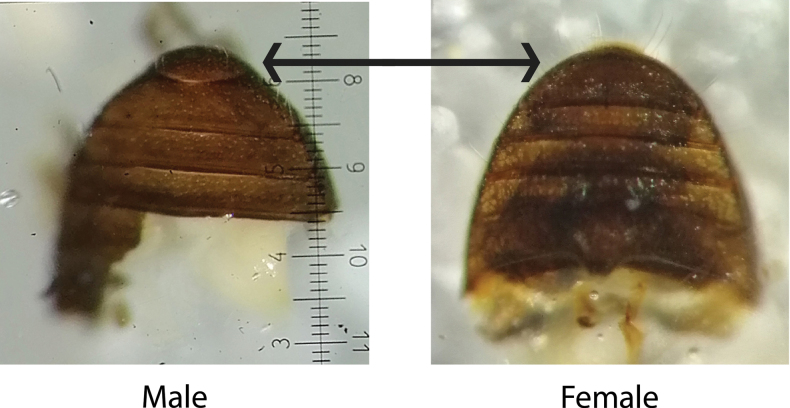
Sexual dimorphism of ventrite in *Cryphalus* spp.

***Sexual dimorphism*.** Males and females can be separated using the last ventrite (Fig. 11), as suggested by (Johnson et al. 2020a). Wood (1982) also suggests that the sexes of several scolytines including *Cryphalus*, can be separated by males having a clearly visible 8^th^ tergite and the females a highly reduced or absent 8^th^ tergite. This character was not examined. We observed some small external differences between males and females. The females (1.77 mm) were slightly larger than males (1.68 mm), and the interstrial setae were overall longer on the females (0.20 mm) compared to the males (0.16 mm). However, there were a considerable overlap between males and females. No clear difference in tubercles or carina on the frons was noticed.

**Male.** The entire aedeagus is ~ 0.6 mm when measured vertically (i.e., from the two points furthest away from each other, Fig. 5). The penis body when seen from above (dorsally) is asymmetrical, evenly broad, and spiralled. Aedeagus apodemes makes up ~ 30% of the entire aedeagus length when measured vertically, they are spiralled and bending downwards. The tegmen is sclerotised and completes a ring around the penis body. It is thin and has two thin ventral apodemes, which are approximately the length of the distance between them (Figs 5, 10C).

**Larvae.** The larvae are described by Kalina (1970).

##### Host plants.

The main hosts of *C.piceae* are *Abies* Mill. and *Picea* Mill. (Escherich 1923; Pfeffer 1995; Wermelinger et al. 2002). In a study on bark beetles which was designed to test their specificity on different conifer hosts, it was found that among *Abies*, *Picea*, *Pinus* L., and *Cupressus* L., *C.piceae* preferred *Abies* and *Picea* (Chararas et al. 1982).

##### Distribution.

*Cryphaluspiceae* is found in Europe: Austria, Bosnia-Herzegovina, Bulgaria, Belarus, Croatia, Czechia, France, Germany, Greece, Hungary, Italy, Latvia, Lithuania, Macedonia, Montenegro, Poland, Romania, Slovakia, Slovenia, Spain, Switzerland, Ukraine, Russia: Central European territory, South European Territory. North Africa: Algeria. Asia: Japan, North Korea, South Korea, Turkey, Russia: Far East, China: North East Territory (Knížek 2011; Alonso-Zarazaga et al. 2023).

*Cryphaluspiceae* has a more southerly distribution. Until now the northernmost record was Denmark (Justesen et al. 2020), but it was recently collected in southern Sweden (Lindelöw and Jonsell 2022). It is found in most of central and southern Europe where *Picea* and *Abies* are present. It has been mentioned from Algeria (Knížek 2011), and possibly also Morocco (Benhalima et al. 2005). However, the distribution of *C.piceae* may be confused with *C.numidicus* in North Africa and in the Mediterranean in general, because of the similar appearance of the two species. Wood (1992b), Sarikaya and Avci (2011) and Cilbircioğlu and Ünal (2012) have mentioned *C.piceae* from Turkey and in the present study we barcoded individuals from Caucasus (Georgia) (Fig. 8). Further studies are needed to understand the distributional overlap and extent of both species. Similarly, the East Palearctic distribution of *C.piceae* is not sufficiently studied as was highlighted by our molecular data (Fig. 8). It is possible that *C.piceae* is present all the way across Russia and parts of China to Japan and Korea (Inouye and Nobuchi 1957; Wood 1992b), but this needs further clarification. As *Abies* is absent from the European part of Russia, the presence of *C.piceae* in this area, is dependent on reproduction in other conifer hosts. See Fig. 10A for distribution.

##### Bionomics.

During the winter adult *C.piceae* hibernate individually on healthy trees, by excavating short tunnels into the phloem (Justesen et al. 2020). Rarely do they also hibernate as larvae or pupae in dead trees or branches infested earlier in the season (Toper 2002). The hibernating adults begin activity around mid/end of March, depending on temperatures (Justesen et al. 2020). During March and April *C.piceae* aggregates on suitable material and mates. The preferred material is weakened parts of trees, or any smaller branches that have broken off during the winter season and are still relatively fresh. After mating they excavate a nuptial chamber, and the female lays 5–26 eggs (Cerchiarini and Tiberi 1997; Toper 2002). The development time from egg to adult depends on temperature. Most commonly, there are two generations a year plus a sister generation; however, in colder regions *C.piceae* only has one generation a year (Justesen et al. 2020).

##### Economic significance.

The harmful properties of *C.piceae* are discussed in detail in Justesen et al. (2020), but most likely *C.piceae* only colonise very weak or recently dead trees. The low impact on host tree survival during colonisation was also confirmed by Justesen et al. (in press B). However, in areas where *C.piceae* reach very high population densities, their ability to penetrate and overwinter in healthy trees could potentially have negative impacts. This could be through lowered tree growth, as the overwintering beetles cause the tree to invest energy into excreting resin from the penetrations. Additionally, the movement from dead or dying trees to healthy trees, could potentially vector fungal diseases, thereby affecting host tree survival. However, the negative impacts of overwintering beetles remain to be explored (Justesen et al. 2020).

##### Remarks.

The shape and size of the aedeagus is the best character to ensure correct identification. The penis body when seen from above is asymmetric and slightly spiralled in *C.piceae*, and highly asymmetric and spiralled in *C.numidicus*. The entire aedeagus is slightly longer (0.6 mm) and broader in *C.piceae* compared to a shorter (0.5 mm) and thinner aedeagus in *C.numidicus*.

Both Grüne (1979) and Noblecourt and Schott (2004) mentioned that the pronotum is anteriorly constricted (dorsal view) (Grüne 1979) or slightly narrowed in the front (Noblecourt and Schott 2004) in *C.piceae* compared to the rounder pronotal shape of *C.numidicus*. In the original description of *C.numidicus* this character is also mentioned (Eichhoff 1878a). This character was partly confirmed. The comparison between the width of pronotum at the widest compared to the width between apex and summit (Fig. 3), generally showed a more rounded shape in *C.numidicus* compared to *C.piceae*, but with a high degree of overlap.

Noblecourt and Schott (2004) mentioned *C.piceae* with shorter setae on the lateral margins of pronotum and elytra compared to *C.numidicus*. During our examinations we also noticed these hair-like setae were shorter in *C.piceae*. Additionally, we found that the lengths of the interstrial hair-like setae on the elytral declivity are markedly shorter in *C.piceae* (0.13–0.23 mm) compared to *C.numidicus* (0.20–0.38 mm). The longer setae on elytra were also mentioned in the original description of *C.numidicus* (Eichhoff 1878a).

Noblecourt and Schott (2004) and Pfeffer (1995) used size as a good separating character between *C.piceae* (1.1–1.6 mm) and *C.numidicus* (1.3–2 mm), whereas Grüne (1979) measured *C.piceae* (1.1–1.8 mm) to be similar sized to *C.numidicus* (1.2–1.8 mm). The original description by Eichhoff found *C.numidicus* to be between 1.3 and 1.6 mm (Eichhoff 1878a). Our measurements did not find a size difference between *C.piceae* (1.45–1.93 mm) and *C.numidicus* (1.50–1.88 mm), but the 16 measured *C.numidicus* specimens were all collected from the same tree.

#### ﻿ Cryphalus numidicus

Taxon classificationAnimaliaColeopteraCurculionidae

Eichhoff, 1878

F69B98DC-8AAA-55C5-9619-458D60EB06A9

Cryphalus numidicus Eichhoff, 1878a: 385.
Cryphalus numidicus Eichhoff: 1878b: 487.

##### Type material.

According to Horn et al. (1990a), Eichhoff’s bark beetle material was transferred via C. Schaufuss and then again via H. Eggers to the Zoological Museum in Hamburg. According to Wood (1992a), the *C.numidicus* material in Hamburg was destroyed. Contact with the Zoological Museum in Hamburg confirmed that the material (if there) was destroyed (pers. comm. Husemann, November 2018).

##### Neotype designation.

**Figure 12.**
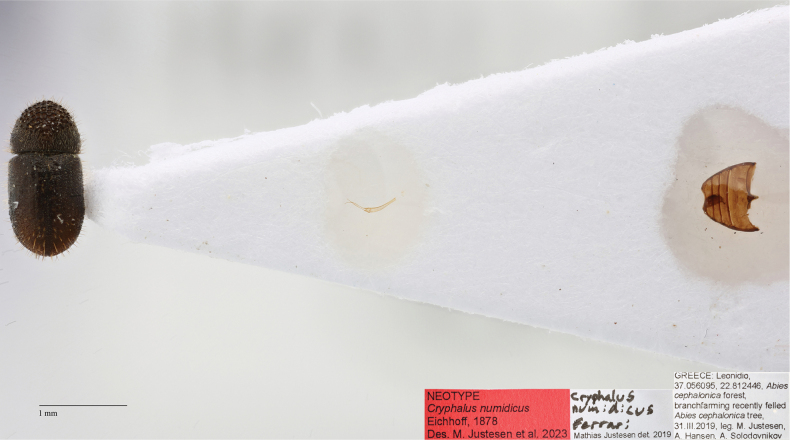
Neotype of *Cryphalusnumidicus*. Stored at NHMD in the entomological collections.

We designate a neotype of *Cryphalusnumidicus* with the express purpose of clarifying the taxonomic status. The original description was based on specimens collected in Greece (Eichhoff 1878a). A male neotype of *Cryphalusnumidicus* (Eichhoff, 1878) was designated (Fig. 12). It was collected on 31 March 2019 in Greece (37°03'21.9"N, 22°48'44.8"E) from an *Abiescephalonica* Loudon tree that had recently fallen. The specimen will be stored at NHMD in the entomological collections. COI sequence (Fig. 8, NUM1 and NUM) are from specimens collected in the same branch as the neotype.

##### Material examined.

55 specimens from a single location in Greece (Table 1) were examined. Morphological measurements were done on 16 specimens from that location. The average results of the measurements are shown in Fig. 2.

##### Diagnosis.

**Figure 13.**
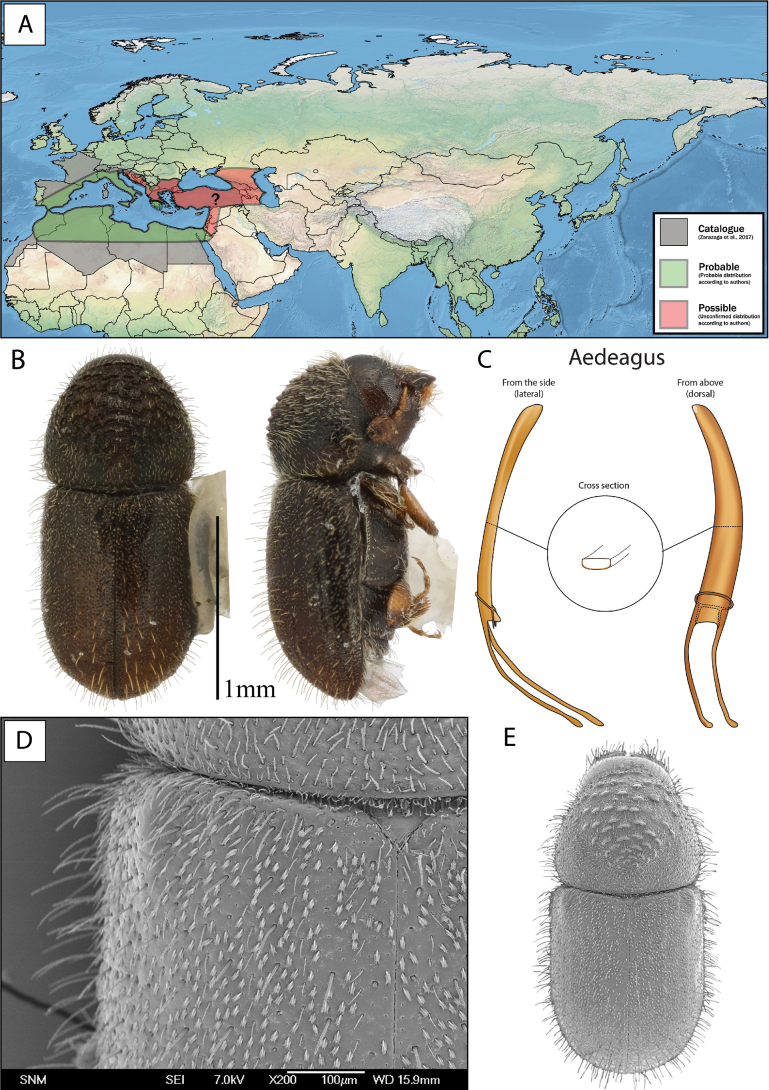
*Cryphalusnumidicus***A** distribution **B** lateral and dorsal view **C** aedeagus **D** SEM, specimen from Leonidio, Greece **E** SEM (43× magnification), specimen from Leonidio, Greece.

This species can be diagnosed from morphologically similar *Cryphalus* in Europe by the combination of circular pronotum, asperities (> 47) on pronotum in almost concentric circles, very long erect interstrial setae on the elytral declivity longer than width of second interstria. For confident identification, the male genitalia is unique. The penis body when seen from above (dorsally) is equally broad and highly asymmetric, spiralled. The entire aedeagus is ~ 0.5 mm in length (Fig. 13B–E).

##### Description.

Length 1.5–1.88 mm, average size 1.68 mm. ***Proportions***: 2.15× as long as wide, elytra 1.30× as long as wide, elytra 1.65× longer than pronotum. ***Antennae***: club with three procurved sutures marked by coarse and long setae. Funiculus with four antennomeres (with pedicel). ***Pronotum***: dark brown to black on both slope and disc. Profile anterior to summit rounded, wider in line with summit. Apical margin with 3–7 asperities, the outer one or two pairs usually smaller, erect setae on entire lateral margins. Anterior slope with > 47 asperities, including the ones on the anterior margin. Disc between 1/4–1/5 the length of pronotum, gently sloping, weakly tuberculate surface texture with a small hair-like seta in each tubercle. Vestiture on declivity and disc hair-like. Suture between pronotum and elytra weakly sinuate. Scutellum: with few trifurcate setae on the margin towards elytra (Fig. 13D). ***Elytra***: usually brown to black, if brown often darker at base, margins equally wide. Surface smooth. Striae with rows of punctures, each puncture with a short hair-like seta, punctures sometimes visible. Interstrial setae long (0.20–0.38 mm) and erect. Interstrial ground vestiture (scales) are serrated, ~ 2–3× as long as wide and translucent brown with a weak iridescence (Fig. 13B, D, E). ***Proventriculus***: sutural teeth of irregular size, confused, in two or more longitudinal rows. Apical teeth extend laterally over the entire segment. Masticatory brush slightly < 1/2 of the proventricular length (Fig. 7).

***Sexual dimorphism*.** Males and females can be separated using the last ventrite (Fig. 11), as suggested by (Johnson et al. 2020a). Wood (1982) also suggests that the sexes of several scolytines including *Cryphalus*, can be separated by males having a clearly visible 8^th^ tergite and the females a highly reduced or absent 8^th^ tergite. This character was not examined. The females (1.74 mm) are on average slightly larger than males (1.66 mm). No clear difference in tubercles or carina on the frons was noticed.

**Male.** The aedeagus is approximately 0.6 mm and the penis body is 0.4 mm, when measured in dorsal view from the two points furthest away from each other (Fig. 5). The penis body when seen from above (dorsally) is highly asymmetrical, equally broad, and clearly spirally twisted. Aedeagus apodemes make up ~ 40% of the entire aedeagus length when measured from dorsal view, they are spiralled and bending downwards. The tegmen is sclerotised and completes a ring around the penis body. It is very thin and has two thin ventral apodemes, which are approximately the length of the distance between them (Figs 5, 13C).

**Larvae.** Nothing is known about the characteristics of the larvae of this species.

##### Host plants.

In North Africa *C.numidicus* is known to occur on *Abiespinsapo* Boiss., *A.numidica* de Lannoy ex Carrière, *Pinushalepensis* Mill. and *Cedrusatlantica* (Endl.) Manetti ex Carrière (Lieutier et al. 2016). In Europe it has been found in *A.pinsapo*, *P.halepensis* (Pfeffer 1995; Lieutier et al. 2016). We collected it from *A.cephalonica*.

##### Distribution.

According to the Palearctic catalogue (Knížek 2011), *C.numidicus* is found in Europe: Bulgaria, France, Greece, Italy, Spain, Switzerland; North Africa: Algeria, Egypt, Libya, Morocco, Tunisia. Asia: Turkey.

Except for Switzerland and Bulgaria, the current distribution of *C.numidicus* is confined to the Mediterranean region, following the distribution of the host species mentioned above. It is unclear if *C.numidicus* occur on *Abiesbornmuelleriana* Mattf., *Abiescilicica* (Antoine & Kotschy) Carrière and *A.nordmanniana* in the East Mediterranean region, or if it is only *C.piceae* that occurs there. We collected it in Greece from *A.cephalonica*. See Fig. 13A for distribution map.

##### Bionomics.

We found adults in mating galleries near Kounoupia in Greece (37°03'21.9"N, 22°48'44.8"E) on 31^st^ March 2019, on an *A.cephalonica* branch, attached to a tree that had fallen during winter, where the bark was still relatively fresh. The branches were recently infested, so activity must have started already in mid-March. This could suggest the possibility of two generations a year. A study by Beghami et al. (2020) showed that *C.numidicus* from Algeria was active in spring, summer, and autumn and that it could reach three generations per year, with two sister broods in early spring and summer and the second generation in mid-September to early November (under favourable weather conditions). Lieutier et al. (2016) stated “The species develops quickly and produces 1–2 generations per year depending on climatic conditions” and “the adults bore very irregular galleries (often invaded by fungi) within the phloem of thin bark on small branches of healthy trees”.

##### Economic significance.

According to Lieutier et al. (2016), *C.numidicus* can cause primary damage if the population density is high and the species can be regarded as a primary and extremely dangerous pest because of its ability to infest and reproduce massively in young healthy trees. The species infests and kills apparently healthy hosts, causing the death of trees (Lieutier et al. 2016). Following Berghami et al. (2020), *C.numidicus* is a “pioneer” species able to establish on relatively freshly cut material, only four months old. *Cryphalusnumidicus* prefers the middle and top part of trees and branches of small diameter. Attacks on cedars are initiated by both *C.numidicus* and *Phloeosinuscedri* C.N.F. Brisout de Barneville, 1883; however, only the latter can attack the crown and the mid-trunk of healthy cedars. After *P.cedri* attacks, *C.numidicus* further impairs cedar defences through massive attacks (Beghami et al. 2020).

##### Remarks.

For discussion on the diagnostic characters separating *C.numidicus* from *C.piceae*, see remarks for the latter species.

#### ﻿ Cryphalus intermedius

Taxon classificationAnimaliaColeopteraCurculionidae

Ferrari, 1867

50BF2E18-A64B-50EC-BCDD-4A638E71D1A2

Cryphalus intermedius Ferrari, 1867: 79.

##### Type material.

According to Horn et al. (1990a), the type material was stored at the Natural History Museum in Vienna. However, no type material was located in that museum (pers. comm. Schillhammer 2018).

##### Material examined.

18 specimens from a single location in Germany (Table 1). Morphological measurements were done on specimens from Germany (7). The average results are shown in Fig. 2.

##### Diagnosis.

**Figure 14.**
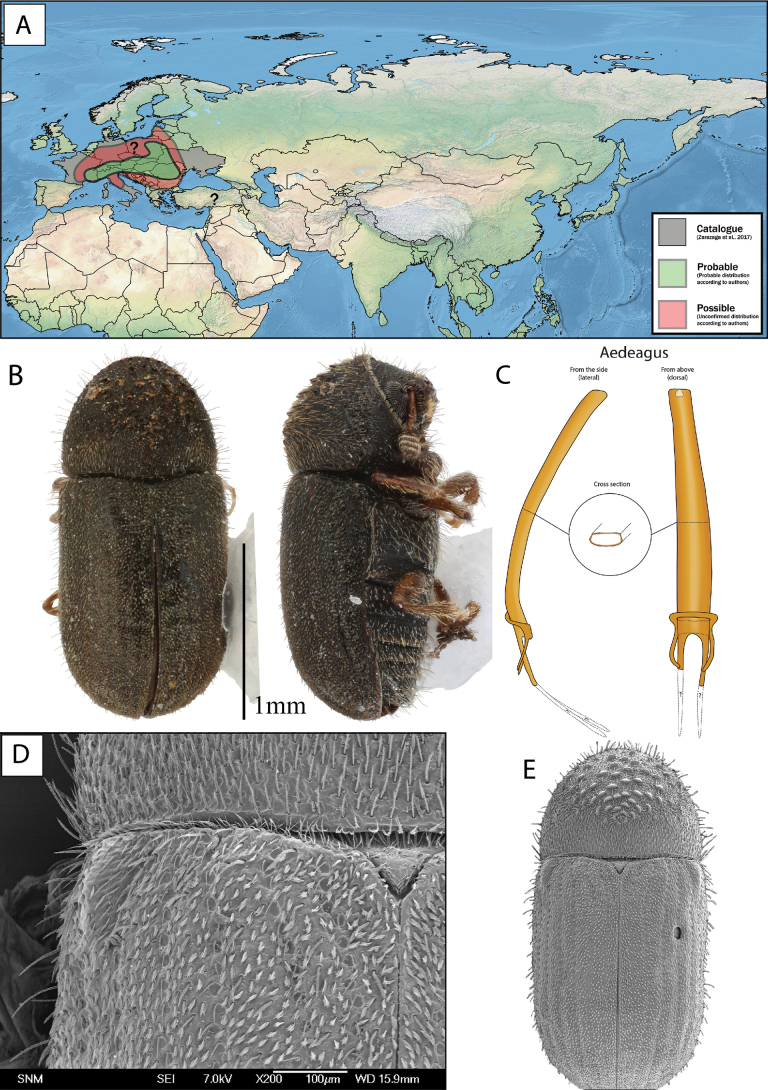
*Cryphalusintermedius***A** distribution **B** lateral and dorsal view **C** aedeagus **D** SEM, specimen from Dresden, Germany **E** SEM (43× magnification), specimen from Dresden, Germany.

This species can be diagnosed from morphologically similar *Cryphalus* in Europe by the combination of size (usually > 1.93 mm), the broadness (elytral width is 0.9–1 mm), interstrial setae on the elytral declivity short (< 0.05 mm), the penis body ~ 0.55 mm in length (Fig. 14B–E).

##### Description.

Length 1.83–2.10 mm, average size 1.98 mm. ***Proportions***: 2.10× as long as wide, elytra 1.29× as long as wide, elytra 1.70× longer than pronotum. ***Antennae***: club with three procurved sutures marked by coarse and long setae. Funiculus with four antennomeres (including pedicel). ***Pronotum***: dark brown to black on both slope and disc. Profile anterior to summit rounded, wider in line with elytral margin. Anterior margin with 2–6 asperities, the outer ones usually smaller, anterior marginal asperities small, erect setae on entire lateral margins. Anterior slope with < 54 asperities, including the ones on the anterior margin. Disc ~1/4 the length of pronotum, gently sloped, weakly tuberculate surface texture with small hair-like setae. Vestiture on declivity and disc hair-like. Suture between pronotum and elytra weakly sinuate. ***Scutellum***: with few trifurcate setae on the margin towards elytra (Fig. 14D). ***Elytra***: usually brown to black, elytral margins equally wide. Surface smooth. Striae with rows of punctures, each puncture with a short hair-like seta, punctures visible and continues on declivity. Interstrial setae short (0.03–0.05 mm) and erect. Interstrial ground vestiture (scales) are serrated, ~ 2–3× as long as wide and translucent brown with a weak iridescence (Fig. 14B, D, E). ***Proventriculus***: sutural teeth of irregular size, confused, in two or more longitudinal rows. Apical teeth extend laterally over the entire segment. Masticatory brush slightly < 1/2 of the proventricular length (Fig. 7).

***Sexual dimorphism*.** Not enough specimens were available to evaluate difference between males and females, but probably the last ventrite can be used to separate males and females externally, as suggested by (Johnson et al. 2020a). Wood (1982) also suggests that the sexes of several scolytines including *Cryphalus*, can be separated by males having a clearly visible 8^th^ tergite and the females a highly reduced or absent 8^th^ tergite. This character was not examined.

**Male.** The aedeagus is probably the biggest among the European species, but due to destroyed apodemes, it was not possible to evaluate the entire length of the aedeagus. The penis body is ~ 0.55 mm, when measured in a dorsal view from the two points furthest away from each other. The penis body when seen from above (dorsally) is almost symmetrical, it is narrowest at the tip and broadens until ~ 1/4 from the base, where it slightly narrows again. Aedeagus apodemes were destroyed during extraction of the aedeagus. The tegmen is sclerotised and completes a ring around the penis body. It is well developed and has two long ventral apodemes, that are ~ 2× the length of the distance between them (Figs 5, 14C).

**Larvae.** Nothing is known about the characteristics of the larvae of this species.

##### Host plants.

It is known from *Larixdecidua* Mill. (Escherich 1923; Grüne 1979; Pfeffer 1995) and *Pinus* (Ferrari 1867; Grüne 1979).

##### Distribution.

According to the Palearctic catalogue (Knížek 2011; Alonso-Zarazaga et al. 2023), *C.intermedius* is found in Europe: Austria, Czechia, France, Germany, Hungary, Italy, Liechtenstein, Poland, Romania, Slovakia, Slovenia, Switzerland, Ukraine.

The current distribution of *C.intermedius* is correlated with the natural range of *Larixdecidua*. However, considering that most surrounding countries outside the natural range of *L.decidua*, have commercial *L.decidua* plantations, it is likely that *C.intermedius* will expand to these plantations in the future. For instance, *C.intermedius* is mentioned from northern Germany in the second supplement to the checklist of German beetles (Köhler 2011). It has also been collected from pine (Ferrari, 1867; Grüne 1979). Grüne (1979) mentions that it occurs in the Alps. See Fig. 14A for distribution map.

##### Bionomics.

The life cycle of the species has not been described in detail. We collected pupae and newly developed adults on 8^th^ of July 2018 from a fallen, but still fresh *Larixdecidua* branch near Dresden, Germany (51°04'26.4"N, 14°28'57.3"E). The number of generations has not been studied in detail, but Trédl (1908) observed newly infested larch branches in July/August and found well developed adults in the same branches in October. Similar to our observations, Trédl (1908) also found well developed adults in July. Wermelinger et al. (2002) collected 18 adult specimens in traps between mid-May and late June. The flight activity in mid-May, the newly developed adults collected in July, and Trédl’s observation of well-developed beetles in October (Trédl 1908) indicate that *C.intermedius* may have two generations per year, as mentioned by Pfeffer (1955).

##### Economic significance.

As far as we know, there has been no reports of this species causing harm to living trees.

##### Remarks.

Several authors found that the elytra of *C.intermedius* is 1.33–1.36× as long as wide and that the body size is ~ 2 mm (Ferrari 1867; Grüne 1979; Pfeffer 1995; Noblecourt and Schott 2004). The seven specimens measured in this study were on average 1.98 mm in body size (1.83–2.10 mm) and elytral proportions were on average 1.32 (1.26–1.38) as long as wide.

Pfeffer′s key (1995) also mentions impressed striae posteriorly on the elytra. This character was not measured but we found it a good diagnostic character.

#### ﻿ Cryphalus asperatus

Taxon classificationAnimaliaColeopteraCurculionidae

(Gyllenhal, 1813)

0D4274CE-5889-55CA-AB5C-232647DA0481

Bostrichus asperatus (Gyllenhal, 1813: 368); designated by Wood 1972: 41.
Bostrichus abietis (Ratzeburg, 1837: 161) (syn: Wood 1972).

##### Type material.

Destroyed during the Second World War together with *C.piceae* type material (see *C.piceae*).

##### Neotype designation.

**Figure 15.**
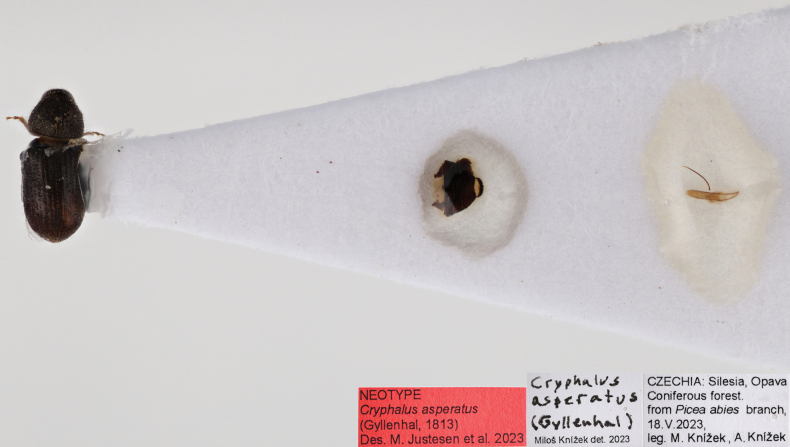
Neotype of *Cryphalusasperatus*. Stored at **NHMD** in the entomological collections.

We designate a neotype of *Cryphalusasperatus* with the express purpose of clarifying the taxonomic status. In the original description, the distribution of *C.asperatus* is mentioned from Upper Silesia (Poland), East Prussia (Poland/Russia), Thuringian Forest (Germany) and Harzen (Germany) and the species is mentioned from *Picea* Mill. (Ratzeburg, 1837). A neotype of *Cryphalusasperatus* (Gyllenhal, 1813) is designated (Fig. 15). It is a male collected on 18/05-2023 from a *Piceaabies* branch collected in Czechia (Silensia) (48°58'20.9"N, 19°35'16.2"E) not far from Upper Silesia. The specimen will be stored at NHMD in the entomological collections.

##### Material examined.

599 specimens from 8 countries in Europe (Table 1) were examined. Morphological measurements were done on 38 specimens from Romania (7), Czechia (12), Slovakia (6), Netherlands (6), and Belgium (7). The results are presented in Fig. 2.

##### Diagnosis.

**Figure 16.**
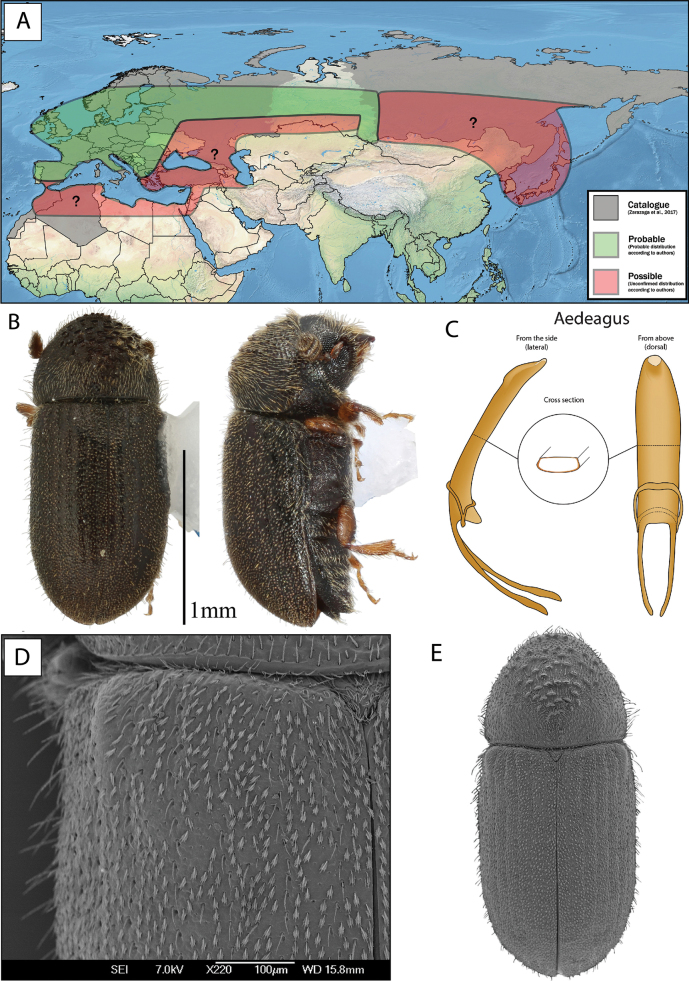
*Cryphalusasperatus***A** distribution **B** lateral and dorsal view **C** aedeagus **D** SEM, specimen from Calibaba, Romania **E** SEM (43× magnification), specimen from Calibaba, Romania.

This species can be diagnosed from similar *Cryphalus* in Europe by the combination of body size usually < 1.75 mm (average 1.61 mm), setae on lateral margin of pronotum clearly shorter between apex and summit compared to setae in line with summit (character 3, Fig. 1), randomly distributed asperities on pronotal declivity (< 50), interstrial setae on the elytral declivity shorter than width of second interstria, often clear elytral striation. For confident identification, extraction of male genitalia is recommended. Penis body when seen dorsally is, aside from the apex, equally broad and is almost bilateral in symmetry. The entire aedeagus ~ 0.5 mm in length (Fig. 16B–E).

##### Description.

Length 1.38–1.90 mm, average 1.61 mm. ***Proportions***: 2.3× as long as wide, elytra 1.5× as long as wide, elytra 1.95× longer than pronotum. ***Antennae***: club with three procurved sutures marked by long setae. Funiculus with four antennomeres (including pedicel). ***Pronotum***: dark brown to black on both slope and disc. Profile anterior to summit triangular to rounded, slightly wider in line with summit. Anterior margin with 2–7 asperities, the outer pair usually smaller, and with erect setae in line with the summit and near apex, usually short or upwards facing in-between. Anterior slope with < 50 asperities, including the ones on the anterior margin. Disc between 1/4–1/5 the length of entire pronotum, gently sloped, weakly tuberculate surface texture with a small hair-like setae in each tubercule. Vestiture on declivity and disc hair-like. Suture between pronotum and elytra weakly sinuate. ***Scutellum***: completely covered with trifurcate hair-like setae (Fig. 16D). ***Elytra***: usually black or dark brown but occasionally light brown, margins parallel and straight. The curvature on the declivity regularly rounded. Surface smooth. Striae often visible as rows of punctures with a short hair-like seta arising from each puncture. Interstrial setae short (0.05–0.08 mm) and erect. Interstrial ground vestiture is serrated, ~ 2–3× as long as wide and translucent brown with a weak iridescence (Fig. 16B, D, E). ***Proventriculus***: sutural teeth of irregular size, confused, in two or more longitudinal rows. Apical teeth extend laterally to < 2/3 of the plate. Masticatory brush slightly < 1/2 of the proventricular length (Fig. 7).

***Sexual dimorphism*.** Males and females can be separated using the last ventrite (Fig. 11), as suggested by (Johnson et al. 2020a). Wood (1982) also suggests that the sexes of several scolytines including *Cryphalus*, can be separated by males having a clearly visible 8^th^ tergite and the females a highly reduced or absent 8^th^ tergite. This character was not examined. No obvious differences in tubercles or carina on the frons was noticed.

**Male.** The aedeagus is ~ 0.5 mm long when measured vertically (i.e., from the two points furthest away from each other). The penis body when seen from above is at the side of the apex, equally broad, and almost bilaterally symmetrical and < 0.4 mm. Aedeagus apodemes makes up ~ 35% of the entire aedeagus length when measured vertically and are more or less straight and bending downwards. The tegmen is sclerotised and completes a ring around the penis body. It is thin and has two ventral apodemes, which are ~ 1/2 the length of the distance between them. The dorsal part of the tegmen ring is narrowest in the middle (Figs 5, 16C).

**Larvae.** For a description of larvae see the work by Ritchie (1918) or Lekander (1968).

##### Host plants.

This species is mentioned in the literature from several conifer genera, but primarily from different *Picea* species (Escherich 1923; Hansen 1956; Lekander et al. 1977; Grüne 1979; Wood 1992b). In a study designed to test the specificity of bark beetles to different conifer hosts, *C.asperatus* was found to prefer *Abies* and *Picea* over *Pinus* and *Cupressus* (Chararas et al. 1982). Ritchie (1918) found *Abies* as a preferred host plant of *C.asperatus*. We collected *C.asperatus* in large numbers from monocultural *Abiesprocera* Rehder plantations in Denmark, which seem to support these data (Justesen et al. 2017; pers. obs. MJJ).

##### Distribution.

According to the Palearctic catalogue (Knížek 2011; Alonso-Zarazaga et al. 2023), *C.asperatus* is found in Europe: Austria, Belgium, Bosnia-Herzegovina, Bulgaria, Belarus, Czechia, Croatia, Denmark, Estonia, Finland, France, Great Britain, Germany, Greece, Hungary, Ireland, Italy, Latvia, Lithuania, Luxemburg, Macedonia, Montenegro, Netherlands, Norway, Poland, Romania, Slovakia, Slovenia, Spain, Sweden, Switzerland, Russia: Central European Territory, North European Territory, South European Territory; North Africa: Algeria, Morocco; Asia: Japan, North Korea, Turkey, Russia: East Siberia, Far East.

The catalogue reported *C.asperatus* in all European countries except Portugal, Ukraine, Moldova, Albania, and Serbia. Nikulina et al. (2015) collected *C.asperatus* in Ukraine and Marković (2013) in Serbia. Considering the natural distribution of *Picea* and *Abies* in Albania, it is unlikely that *C.asperatus* is not present here as well. Studies from Finland (Voolma et al. 2004) and distribution maps from Sweden (Artportalen 2023) and Norway (Artsdatabanken 2023), show that *C.asperatus* is not adapted to the Arctic region. The fact that the catalogue mentions *C.asperatus* from Japan, North Korea, and Eastern Russia needs confirmation. Distribution records from these areas could be erroneous because of similar looking species, e.g., *Cryphalussichotensis* Kurenzov, 1941, *C.saltuarius* or others. More comparative work on the eastern species, similar to that of Johnson et al. (2020b), is necessary to figure out the easternmost extent of *C.asperatus*. Our distribution map follows the note by Mandelshtam (2002) stating that *C.asperatus* is not found east of Altai (Mandelshtam 2002). Records of *C.asperatus* in Morocco and Algeria also need confirmation, especially considering the one specimen (1.28: Georgia, Tlughi) from Georgia, which is 5.6% different from the European populations (Fig. 8). A larger sampling in and around Georgia, including in-depth morphological study are needed to elucidate the relationship and establish if these specimens represent a separate species or just intraspecific variation. See distribution illustrated in Fig. 16A.

##### Bionomics.

During winter *C.asperatus* can hibernate as adults, larvae, pupae and more rarely as eggs (Ritchie 1918; Pfeffer 1955). It hibernates underneath the bark of infested material (Ritchie 1918; pers. obs. MJJ). Flight activity can start already in March (Ritchie 1918; data from Wermelinger et al. (2002); pers. obs. MJJ). Comparable unpublished data from Denmark showed that *C.asperatus* became active a few weeks earlier than *C.piceae*. During the period from March to May *C.asperatus* aggregates on suitable material and mates. The males will try to mate with as many females as possible, and after mating the males will excavate a nuptial chamber. Males of *C.asperatus* display a very distinct preference for branch nodes, and often you find branches where only nodes are inhabited (Ritchie 1918; Justesen et al. 2017). This preference is so evident that it was mentioned in the original description by Ratzeburg (1837). The preferred material seems to be moist and shaded branches, compared to sun exposed dry branches (Ritchie 1918; pers. obs. MJJ). Compared to the other European *Cryphalus* species, *C.asperatus* can target relatively old/decomposed material but can also be found in recently fallen branches. Once the males complete their nuptial chamber, the female will lay 14–24 eggs (Ritchie 1918). The development from egg to adult is variable depending on temperature, type of material, the position of the material (sun-exposed) and the time of egg-laying (Ritchie 1918). According to Ritchie (1918) two generations per year is unlikely, but he mentions the possibility of a sister generation. Grüne (1979) and Pfeffer (1955) suggested two generations per year. In an unpublished study from Denmark, 90 *Abiesprocera* branches were cut and placed as bait in an *A.procera* plantation in the spring. Six branches were then collected every second week and evaluated for the presence of various life stages of *C.asperatus*. These results suggested one generation. Based on the above information, *C.asperatus* most likely has two generations under ideal conditions and only one in colder climates.

##### Economic significance.

In older literature *C.asperatus* is described as a possible harmful pest (Eichhoff 1881; Nüßlin 1905; Bodenheimer 1958). However, as already mentioned by Ritchie (1918) and Hansen (1956), these reports seem unlikely. A recent study looking at Norway spruce seedlings weakened by transport, found *C.asperatus* as a potential problem (Fiala and Holuša 2021). Our observations of *C.asperatus* support that this species is a harmless species not able to kill or weaken trees.

##### Remarks.

Differences between *C.asperatus* and *C.saltuarius*.

The shape and size of the aedeagus is the best character to separate the two species. The penis body when seen dorsally is equally broad in *C.asperatus*, but broadest one quarter down from the apex and then becomes increasingly narrow towards the base in *C.saltuarius*. The entire aedeagus is longer (~ 0.7 mm) in *C.saltuarius* compared to *C.asperatus* (~ 0.5 mm).The size difference between *C.asperatus* and *C.saltuarius* is commonly highlighted and the following lengths were reported for *C.asperatus*: 1.75 mm average (Ritchie 1918), 1.2–1.7 mm (Grüne 1979; Pfeffer 1995; Noblecourt and Schott 2004), 1.2–1.8 mm (Hansen 1956), and 1.3–1.8 mm (Spessivtseff 1922). We measured 38 *C.asperatus* specimens and found a range of 1.38–1.90 mm but, besides one noticeably larger specimen, the remaining 37 specimens were all < 1.75 mm. The following lengths were reported for *C.saltuarius*: 1.5–2 mm (Spessivtseff 1922; Hansen 1956; Grüne 1979; Pfeffer 1995) and 1.5–2.2 mm (Noblecourt and Schott 2004). We measured 25 specimens of *C.saltuarius* to 1.73–1.98 mm, with eight specimens lying between 1.73–1.75 mm. These measurements confirm that body size often is a reliable character, but also highlights that overlap occurs.

**Figure 17.**
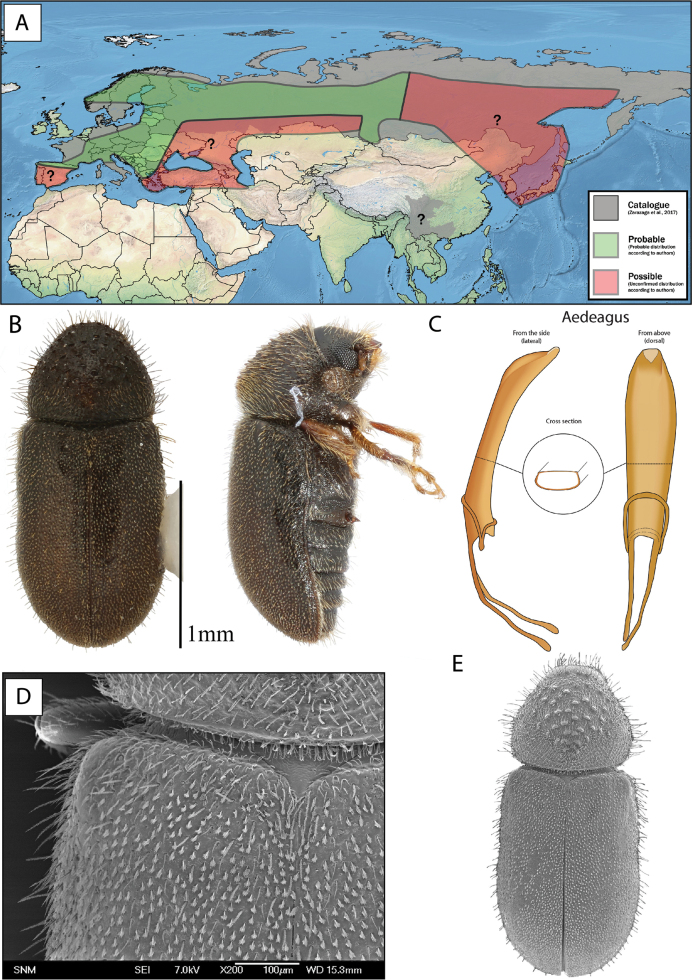
*Cryphalussaltuarius***A** distribution **B** lateral and dorsal view **C** aedeagus **D** SEM, specimen from Østby, Norway **E** SEM (43× magnification), specimen from Østby, Norway.

We found that *C.saltuarius* specimens usually had longer and perpendicularly erect setae along the margins of pronotum (Figs 1, 17B, E), whereas most *C.asperatus* only had erect setae in line with the summit and near apex, and then short and sometimes upwards facing setae in-between apex and summit (Fig. 16B, E). It should be mentioned that authors have observed old *C.saltuarius* museum specimens lacking these setae. The scutellum of *C.asperatus* is covered in trifurcate hair-like setae, whereas *C.saltuarius* only has these hairs along the elytral margin of scutellum (Figs 16D, 17D); however, this character requires high magnification and was therefore not included in the key.

Several keys mention that the striae in *C.asperatus* are clearer (Fig. 16B, D, E) compared to more indistinct striae in *C.saltuarius* (Fig. 17B, D, E) (Ritchie 1918; Spessivtseff 1922; Hansen 1956; Grüne 1979; Pfeffer 1995; Noblecourt and Schott 2004). Generally, we could confirm this tendency (Fig. 4), but nine of the 25 *C.saltuarius* specimens had clearer striation, which could be confused with *C.asperatus* specimens with less distinct striation.

Noblecourt and Schott (2004) used the shape of the elytral declivity to separate *C.asperatus* (regular curvature) from *C.saltuarius* (flattened on the declivity). Our studies also found this tendency, but with a slight overlap (Fig. 4).

Most keys include proportional differences as a good character to separate the species. Noblecourt and Schott (2004) found *C.asperatus* was 2.3× longer than wide and *C.saltuarius* 2× longer than wide. Our results (Fig. 2) showed a slight tendency of *C.asperatus* being comparably longer than wide, but with a very high degree of overlap between the species. Grüne (1979) and Pfeffer (1995) found that the elytra of *C.asperatus* was 1.5–1.57× as long as wide, whereas *C.saltuarius* was 1.6–1.67× as long as wide. Our measurements had a large overlap in the proportional difference of elytra. Therefore, we did not find proportions as a good character. It should be noted that we measured elytral width as in Fig. 1, across the scutellum. Measurements 1/3 down from the basal border of elytra could slightly increase the width measurements, resulting in less similar proportions.

#### ﻿ Cryphalus saltuarius

Taxon classificationAnimaliaColeopteraCurculionidae

Weise, 1891

F463B08E-EEE1-57BF-A6F5-9E4FCB751497

Cryphalus saltuarius Weise, 1891: 336.
Bostrichus asperatus Ratzeburg, 1837: 163; suggested by Eichhoff (1878b: 137).
Cryphalus scriba de Gozis, 1886: 31; nomen oblitum (Knížek 2017).

##### Type material.

*Cryphalussaltuarius* was first described by Gyllenhal in 1813, under the name *Bostrichusasperatus*. According to Horn et al. (1990a), Gyllenhal’s material was stored at the Zoological Museum in Uppsala. Wood (1967) located the type series originally used by Gyllenhal, at the Zoological Museum in Uppsala and designated a lectotype. Additionally, three paralectotypes were stored in the Germar collection at the Museum für Naturkunde in Berlin (Wood 1972). Pictures of type material is shown in Justesen et al. (in press A).

##### Material examined.

98 specimens from various locations in Sweden and Norway (Table 1) were examined. Morphological measurements were done on 25 specimens from Norway (20) and Sweden (5). The results are shown in Fig. 2.

##### Diagnosis.

This species can be distinguished from similar *Cryphalus* in Europe by the combination of body size usually > 1.75 mm (average 1.82 mm), randomly distributed asperities on pronotal declivity (< 54), erect interstrial setae on the elytral declivity shorter than width of second interstria, setae on lateral margin of pronotum as long or only slightly shorter between apex and summit compared to setae in line with summit (character 3, Fig. 1), often obsolete elytral striation, elytral declivity often more or less flattened. For confident identification, the male genitalia is unique, because the penis body when seen from above (dorsally) is broadest one quarter down from the apex and then becomes increasingly narrow towards the base, it is almost bilateral in symmetry. The entire aedeagus is ~ 0.7 mm in length (Fig. 17B–E).

##### Description.

Length 1.73–1.98 mm, average size 1.82 mm (lectotype 1.85 mm). ***Proportions***: 2.28× as long as wide, elytra 1.46× as long as wide, elytra 1.96× longer than pronotum. ***Antennae***: club with three procurved sutures marked by coarse and long setae. Funiculus with four antennomeres. ***Pronotum***: dark brown to black on both slope and disc. Profile anterior to summit almost triangular but slightly rounded, slightly wider in line with summit. Anterior margin with 2–5 asperities, the outer pair usually smaller, erect setae on entire lateral margins of pronotum. Anterior slope with < 50 asperities, including the ones on the anterior margin (lectotype has 44). Disc between 1/4–1/5 the length of pronotum, gently sloped, weakly tuberculate surface texture with small hair-like setae in each tubercule. Vestiture on declivity and disc hair-like. Suture between pronotum and elytra weakly sinuate. ***Scutellum***: small with almost no setae (Fig. 17D). ***Elytra***: usually dark brown or black but sometimes pale brown, margins widest 2/3 down from pronotal edge. The curvature on declivity irregularly rounded. Surface smooth. Striae usually only visible as rows of short hair-like setae. Interstrial setae short (0.05–0.08 mm) and erect. Interstrial ground vestiture is serrated, ~ 2× as long as wide and translucent brown with a weak iridescence (Fig. 17 B, D, E). ***Proventriculus***: sutural teeth of irregular size, confused, in two or more longitudinal rows. Apical teeth do not extend laterally on the plate. Masticatory brush slightly < 1/2 of the proventricular length (Fig. 7).

***Sexual dimorphism*.** Males and females can be separated using the last ventrite (Fig. 11), as suggested by (Johnson et al. 2020a). Wood (1982) also suggests that the sexes of several scolytines including *Cryphalus*, can be separated by males having a clearly visible 8^th^ tergite and the females a highly reduced or absent 8^th^ tergite. This character was not examined. Females were on average slightly larger than males, 1.79 mm and 1.84 mm, respectively. No clear difference in tubercles or carina on the frons was noticed.

**Male.** The aedeagus is ~ 0.7 mm long when measured vertically (i.e., from the two points furthest away from each other). The penis body when seen from above (dorsally) is almost bilaterally symmetrical and is broadest 1/4 from the apex and then becomes increasingly narrow towards the base. It is > 0.4 mm. Aedeagus apodemes makes up ~ 40% of the entire aedeagus length when measured vertically, more or less straight and bending downwards. The tegmen is sclerotised and completes a ring around the penis body. It is thin and has two ventral apodemes, which are ~ 1/2 the length of the distance between them (Figs 5, 17C).

**Larvae.** For a description of larvae see the work by Lekander (1968).

##### Host plants.

It is mostly mentioned from various *Picea* species (Spessivtseff 1922; Wood 1992b; Pfeffer 1995; Peltonen and Heliövaara 1998; Wermelinger et al. 2002), but has also been mentioned on *Abies*, *Pinus* (Pfeffer 1995), and *Juniperus* L. (Negru 1958).

##### Distribution.

According to the catalogues (Knížek 2011; Alonso-Zarazaga et al. 2023), *C.saltuarius* is found in Europe: Austria, Bulgaria, Belarus, Czechia, Denmark, Estonia, Finland, France, Germany, Hungary, Italy, Liechtenstein, Montenegro, Norway, Poland, Slovakia, Sweden, Switzerland, “Caucasus”, Russia: Central European Territory, North European Territory; Asia: China: Guangxi, Sichuan, Yunnan, Russia: West and East Siberia, Far East, West Siberia.

*Cryphalussaltuarius* is a rather common species in the Arctic regions of Scandinavia (Lekander et al. 1977). It is often regarded as rare in central Europe (Negru 1958) and it was only recently discovered in France (Noblecourt and Schott 2004). *Cryphalussaltuarius* has a boreo-montane distribution (Pfeffer 1995). The species is most likely present in most mountain ranges in Europe, wherever the host species is present including, for example, countries such as Ukraine (Nikulina et al. 2015), Romania (Nitzu and Olenici 2009), and Slovenia (Jurc 2003) are not mentioned in the catalogue. According to the palearctic catalogues (Knížek 2011; Alonso-Zarazaga et al. 2023) the species is present across Russia and even in some southern provinces of China. Although Kurenzov (1941), Krivolutskaya (1996) and Yanovskij (1999) reported the species from Far East Russia, Mandelshtam (2002) did not locate any specimens to confirm these records. Like *C.asperatus* there is not enough information about the presence of this species in the eastern Palearctic and Caucasus. The records from the Chinese provinces are interesting if confirmed, but they may be based on misidentification of another species and need confirmation. See distribution illustrated in Fig. 17A.

##### Bionomics.

There are no specific studies on the life cycle of *C.saltuarius*. Peltonen and Heliövaara (1998) wrote that “*C.saltuarius* disperses during the midsummer and breeds in spruce under thin bark, e.g., in snow-broken spruce tops, branches and smaller trees”. Lekander et al. (1977) found that *C.saltuarius* had a two-year generation cycle, with swarming in June and hibernation as larvae. These overwintering larvae complete their development in June the following year. The newly developed beetles then hibernate. Adults and larvae were observed in Østby, Norway on 22 October 2018 from a small *Piceaabies* tree which fell in spring 2018. We hatched 72 specimens from a small branch sampled from the tree. In the Wermelinger et al. (2002) study, 62 specimens of *C.saltuarius* were caught by window traps. More than 90% of these specimens were caught between end of April and beginning of June, indicating a different life cycle than described above, possibly due to the different temperatures in central Europe or confusion with the very similar *C.asperatus*. Pfeffer (1955) mentions two generations per year, and only one generation in higher altitude sites.

##### Economic significance.

In Lekander et al. (1977), *C.saltuarius* is described as a secondary pest species, which under the right conditions can attack weakened, but still living spruce trees. Probably the harmfulness of this species is like *C.piceae*, in that only very weakened trees are attacked. Therefore, this species should not without question, be regarded as a pest, despite being abundant in recently dead trees.

##### Remarks.

For discussion on the diagnostic characters separating *C.saltuarius* from *C.asperatus*, see remarks for the latter species.

## Supplementary Material

XML Treatment for Cryphalus piceae

XML Treatment for Cryphalus numidicus

XML Treatment for Cryphalus intermedius

XML Treatment for Cryphalus asperatus

XML Treatment for Cryphalus saltuarius

## ﻿Additional information

### Author ORCIDs

Mathias Just Justesen https://orcid.org/0000-0002-5252-7045

Aslak Kappel Hansen https://orcid.org/0000-0003-2089-7233

Milos Knížek https://orcid.org/0000-0003-3221-9448

Alexey Solodovnikov https://orcid.org/0000-0003-2031-849X

Hans Peter Ravn https://orcid.org/0000-0001-5090-3273

### Data availability

All of the data that support the findings of this study are available in the main text or Supplementary Information.

## Supplementary materials

Supplementary material 1 Lateral and dorsal photographs of male genitalia of five European *Cryphalus* species Data type pdf https://binary.pensoft.net/file/903302This dataset is made available under the Open Database License (http://opendatacommons.org/licenses/odbl/1.0/). The Open Database License (ODbL) is a license agreement intended to allow users to freely share, modify, and use this Dataset while maintaining this same freedom for others, provided that the original source and author(s) are credited.Mathias Just Justesen, Aslak Kappel Hansen, Milos Knížek, Åke Lindelöw, Alexey Solodovnikov, Hans Peter Ravn
