# Supplementary material for: ﻿Taxonomic reappraisal of the European fauna of the bark beetle genus Cryphalus (Coleoptera, Curculionidae, Scolytinae)
Source: Zookeys. 2023 Sep 8;1179:63–105. doi: 10.3897/zookeys.1179.101388 (PMC10504635; doi:10.3897/zookeys.1179.101388)
Supplement: Supplementary material 1 — Lateral and dorsal photographs of male genitalia of five European Cryphalus species [file zookeys-1179-063_article-101388__-s001.pdf]

Supplementary, File 1:

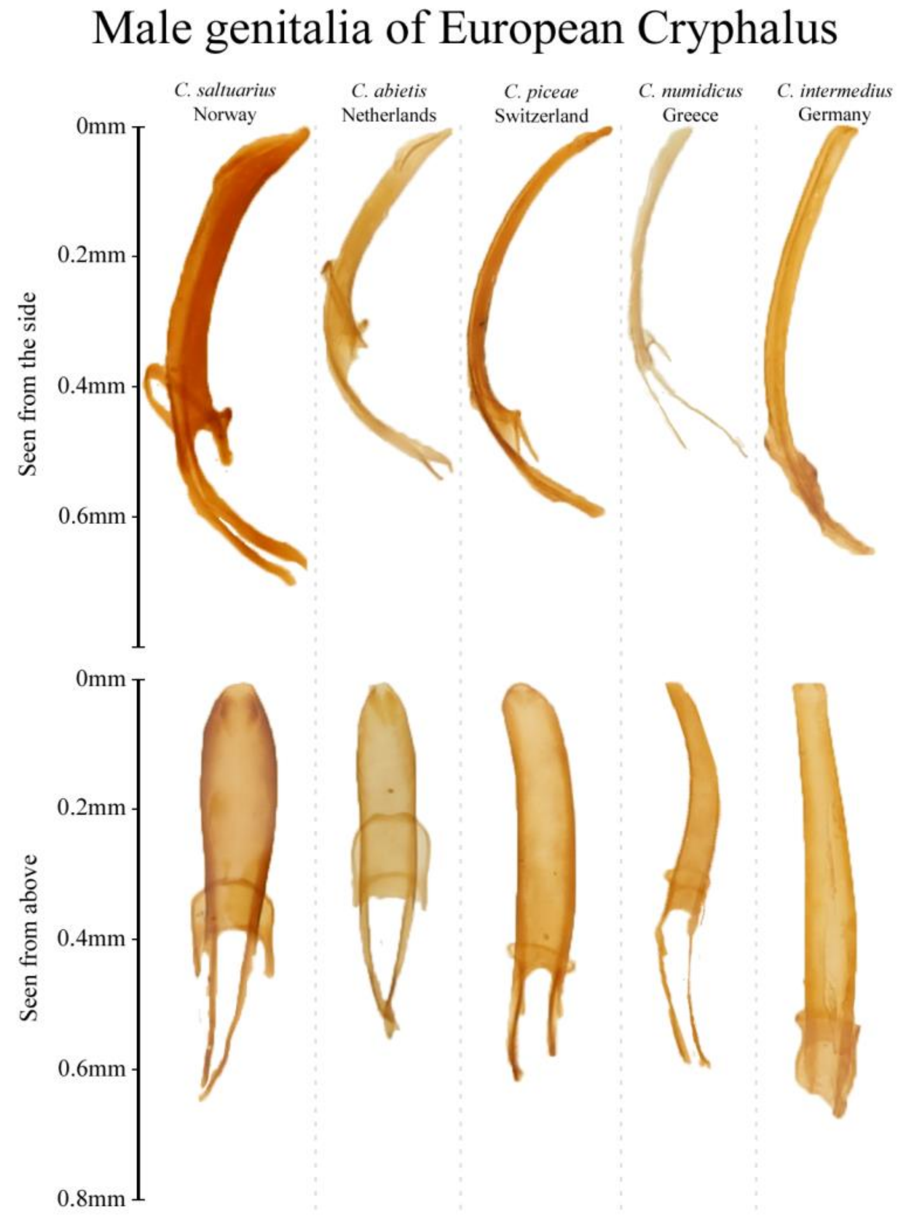

Foto 1: Lateral and dorsal fotos of male genitalia of five European *Cryphalus* species.
